# The Role of Working Memory for Cognitive Control in Anorexia Nervosa versus Substance Use Disorder

**DOI:** 10.3389/fpsyg.2017.01651

**Published:** 2017-09-22

**Authors:** Samantha J. Brooks, Sabina G. Funk, Susanne Y. Young, Helgi B. Schiöth

**Affiliations:** ^1^Functional Pharmacology, Department of Neuroscience, Uppsala University Uppsala, Sweden; ^2^Department of Psychiatry and Mental Health, University of Cape Town Cape Town, South Africa; ^3^Department of Psychiatry, Stellenbosch University Bellville, South Africa

**Keywords:** working memory, cognitive control, anorexia nervosa, substance use disorder, neuropsychology, neural, genetic, working memory training

## Abstract

Prefrontal cortex executive functions, such as working memory (WM) interact with limbic processes to foster impulse control. Such an interaction is referred to in a growing body of publications by terms such as cognitive control, cognitive inhibition, affect regulation, self-regulation, top-down control, and cognitive–emotion interaction. The rising trend of research into cognitive control of impulsivity, using various related terms reflects the importance of research into impulse control, as failure to employ cognitions optimally may eventually result in mental disorder. Against this background, we take a novel approach using an impulse control spectrum model – where anorexia nervosa (AN) and substance use disorder (SUD) are at opposite extremes – to examine the role of WM for cognitive control. With this aim, we first summarize WM processes in the healthy brain in order to frame a systematic review of the neuropsychological, neural and genetic findings of AN and SUD. In our systematic review of WM/cognitive control, we found *n* = 15 studies of AN with a total of *n* = 582 AN and *n* = 365 HC participants; and *n* = 93 studies of SUD with *n* = 9106 SUD and *n* = 3028 HC participants. In particular, we consider how WM load/capacity may support the neural process of excessive epistemic foraging (cognitive sampling of the environment to test predictions about the world) in AN that reduces distraction from salient stimuli. We also consider the link between WM and cognitive control in people with SUD who are prone to ‘jumping to conclusions’ and reduced epistemic foraging. Finally, in light of our review, we consider WM training as a novel research tool and an adjunct to enhance treatment that improves cognitive control of impulsivity.

## Introduction

**FIGURE 1:**
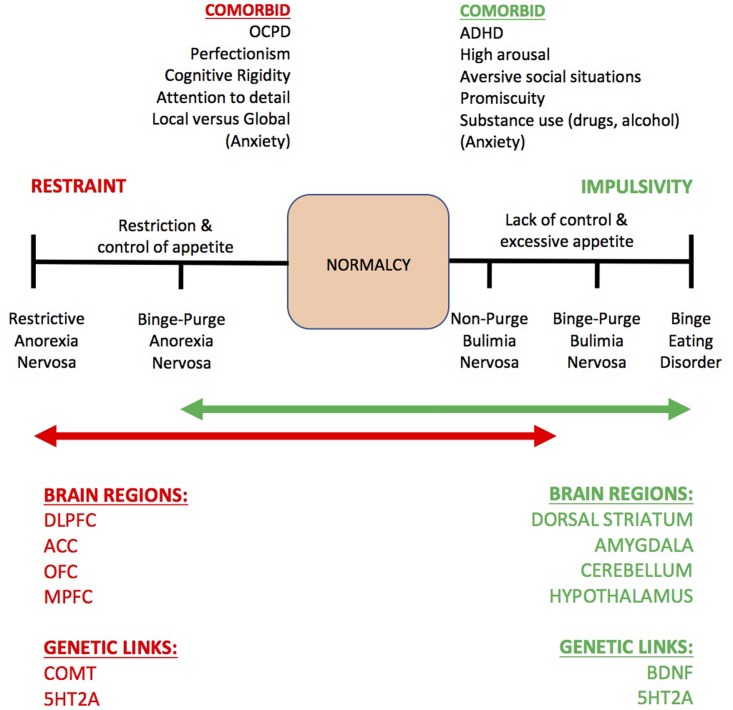
The impulse control spectrum model of eating disorders by Brooks et al. (2012b, 2016). This model describes the common comorbid neuropsychological traits, some neural and genetic markers of restraint versus impulsivity, as related to eating behavior. SUD is regarded, based on studies reviewed in this paper, to correspond to similar processes as binge eating disorder (Volkow and Baler, 2015), at the opposite end of the spectrum to restricting anorexia nervosa. Normalcy – or normal appetite/impulse control – is deemed to be in the middle of the spectrum. OCPD, obsessive-compulsive personality disorder; DLPFC, dorsolateral prefrontal cortex; OFC, orbitofrontal cortex; MPFC, medial prefrontal cortex; ACC, anterior cingulate cortex; COMT, catechol-*O*-methyl-transferase; 5HT2A, 5-hydroxy-tryptophan-2A (serotonin receptor 2A gene); BDNF, brain derived neurotrophic factor. Of note: anxiety is hypothesized to be experienced when the system is imbalanced.

‘*Can we learn about the treatment of substance use disorder (SUD) from the neural correlates of anorexia nervosa (AN)?*’, is a question that has recently been debated in line with a spectrum model of impulse control (**Figure 1**) (Brooks et al., 2012b; Brooks, 2016). The previous articles debated a model where healthy impulse control (‘normalcy’) is in the middle, excessive control (e.g., AN) is at one extreme, and lack of control (e.g., binge eating, SUD) is at the other extreme (Volkow and Baler, 2015). The previous articles emphasized a theory emerging in the eating disorder literature that increased working memory (WM) capacity may underlie excessive cognitive control in AN. Increased WM capacity, according to the previous articles, may manifest as cognitive rumination, excessive attention to detail (e.g., ‘epistemic foraging’), local versus global cognitive processing and strategies that remain rigidly in mind for increasingly complex and detailed eating disordered thoughts (Kothari et al., 2013) toward future goals that are not certain to be achieved (e.g., about shape, weight, eating, and body image). The previous articles further suggested that increased WM capacity may contribute to altered neurophysiology and the maintenance of restraint of appetite in AN (which also appears protective against the development of SUD, see Kaye et al., 2013b). At the opposite extreme of an impulse control spectrum, it is proposed that people with SUD have a reduced WM capacity and a lack of cognitive control over impulses to consume substances and a proneness to ‘jumping to conclusions,’ coinciding with disrupted dopaminergic transmission in the mesolimbic pathway (Everitt and Robbins, 2016). Similarly, adults who have been consistently obese for 5 years compared to those who have been consistently lean show deficits in WM performance and smaller prefrontal cortex brain volume (Brooks et al., 2013). Intriguingly, cognitive control of appetite in AN can itself become rewarding, rigid and deeply ingrained, switching from deliberative and recreational (e.g., occasional dieting) to habitual and compulsive, hijacking dopaminergic networks in the brain akin to the addiction process (Everitt, 2014; O’Hara et al., 2015).

It is against this background that the present systematic review of AN and SUD aims to progress the theoretical perspective posed by the impulse control spectrum model (ibid), with a structured review of the neurobiological substrates of WM processes (e.g., neuropsychological, neural, and genetic). In line with the aim of systematically reviewing, the literature for comparison of AN and SUD to support the previous theoretical debate (Brooks et al., 2012b; Brooks, 2016) we provide an introduction summarizing the neural processes of normal WM proposed by model of Baddeley and Hitch (1974). To progress the basic tenets of the WM model, we additionally summarize two contemporary theories related to WM, namely Global Workspace Theory (Baars et al., 2013) and Bayesian Probabilistic Interference (Nilsson, 1986; Friston, 2012). After introducing some of the latest neuroscientific theories of WM and processes related to cognitive control, we then systematically review findings regarding WM and cognitive control in AN and SUD – populations that underpin the major tenets of the impulse control spectrum model (ibid). We do this to examine and contrast how WM might play a role in variations of cognitive control in these differing populations. Finally, we end by suggesting potential mechanisms underlying WM training as a novel research tool and an approach to improving treatment for impulse control disorders. For a summary of systematically reviewed publications of AN and SUD, see Supplementary Tables S1, S2.

### Inclusion/Exclusion Criteria for Systematic Review of AN and SUD Studies

See Supplementary Table S3 for PRISMA diagram of the systematic review flowchart.

To conduct a structured review of the role of WM in cognitive control associated with AN and SUD we used the following search inclusions and exclusions as below. Of note, we do not provide a systematic review of WM and cognitive control in healthy controls, given that since the introduction of the WM model by Baddeley and Hitch (1974), there has been an explosion of studies, theories and opinions using various terms synonymous with cognitive control, including *cognitive inhibition, affect regulation, self-regulation, top-down control and cognitive–emotion interaction*. As such, some contemporary theories of healthy WM are introduced, and thereafter studies are systematically reviewed that measure WM in AN and SUD in line with the opposing extremes of the impulse control model described in **Figure 1**.

Consulting PubMed, Medline, Science Direct, and manual searches of publication reference lists, we used the following search terms for inclusion in the systematic review of AN and SUD studies: *anorexia AND working memory anorexia nervosa AND working memory; anorexia AND working memory AND cognitive control anorexia nervosa AND working memory AND cognitive control; substance use disorder AND working memory; substance use disorder AND working memory AND cognitive control*. Of note, we only included AN and SUD (and not, for e.g., ‘eating disorders’ or ‘addiction’) to search for those specific populations we are considering at the extremes of an impulse control spectrum model. Our aim is to systematically review the role of WM in cognitive control in AN and SUD. Exclusion criteria were: systematic reviews/meta-analysis and theoretical/opinion/perspective articles (although some of these articles are referred to in our discussions of the empirical work); articles not written in English; articles measuring other executive functions but not WM; articles where AN/SUD was comorbid/secondary to major mental/neurological disorder (e.g., schizophrenia/cognitive decline associated with seropositive HIV/fetal alcohol syndrome/bipolar disorder); articles within the last decade – January 2010 – present: August 2017 (to include recent neuroscientific literature). As such, we found *n* = 15 studies that directly measured WM and cognitive control in AN, and *n* = 93 studies in SUD.

In order to contextualize the findings of the systematic review of studies examining WM and its role in cognitive control in AN and SUD, we first provide an overview of theories and some empirical studies in the healthy human brain.

## Theories of Working Memory and Cognitive Control in the Healthy Human Brain

The major suggestion, or ‘red line’ throughout this article is that WM capacity may not be limited, as traditionally posited by Miller (1956) by the “*magical number seven, plus or minus two*” items that can be remembered. Rather, that WM capacity can ultimately be widened, deepened or more flexibly developed for the improved cognitive control of impulsivity with the employment of repetitive WM strategies. It has been previously shown that control of impulsivity – by way of keeping future goals in mind when making decisions in the face of internal and/or external salient, often rewarding/arousing distractions – Yantis (2000) is fostered by dual processes associated with executive functions such as WM (Bechara, 2005). Repetitive and increasingly complex or difficult engagement of WM we propose, may alter neural processes that ultimately alter cognitive control of impulsivity in those who would be broadly considered to have impulse control disorders – such as those with AN (excessive control) or SUD (weakened control). This thinking is in line with the dual process model of cognitive control, whereby cold, slower, reflective, top-down, conscious, explicit executive functions are utilized to exert moderation over hot, faster, reflexive, bottom-up, unconscious, implicit arousal responses (Bechara, 2005; Kahneman, 2011; Sofuoglu et al., 2016). It has been known for decades that a threshold of prefrontal cortex activation is needed for effective modulation of bottom-up processes and is associated with WM (e.g., Goldman-Rakic, 1995, 1998). The top-down cognitive control of such bottom-up processes could be regarded as “free won’t,” or conscious veto (Wegner, 2002) in response to an automatic *readiness potential* in the brain (perhaps akin to impulsivity) occurring up to half a second prior to conscious experience (Libet, 1985). The employment of a conscious veto, we argue, might be supported by WM processes that translate into action tendencies (Eimer and Schlaghecken, 2003), behavioral control and memory retrieval suppression (Anderson et al., 2016). However, WM processes, which we consider in this article, may be dysfunctional and lead to psychiatric disorders such as AN and SUD.

### The Working Memory Model (Baddeley and Hitch, 1974)

**FIGURE 2:**
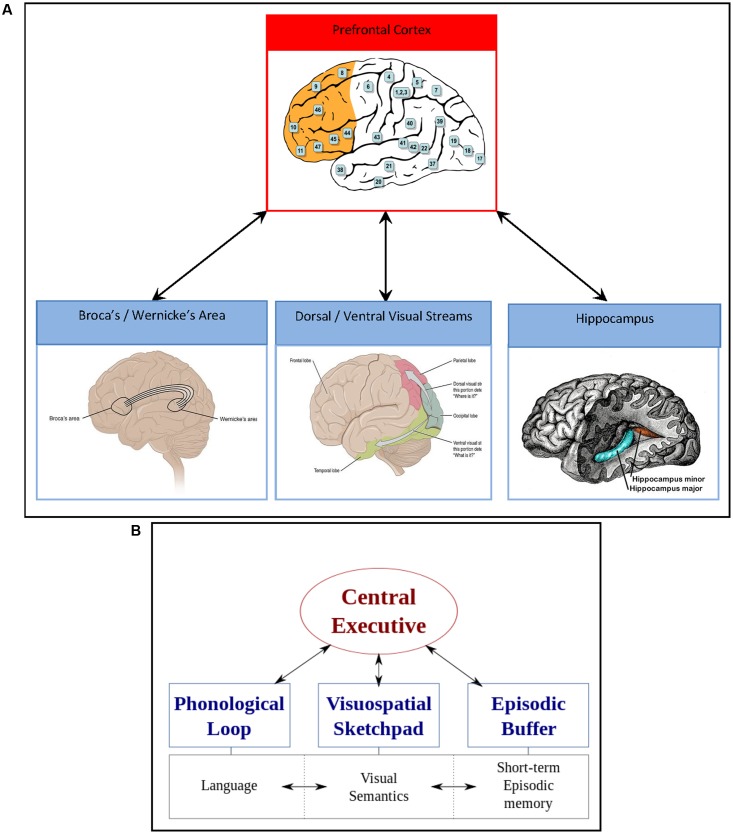
**(A)** Neurobiological depiction of the WM model. Red square represents the prefrontal cortex (in orange) and the central executive; left blue square represents the language network underling the phonological loop, namely speech production (Broca’s area in the frontal cortex) and speech comprehension (Wernicke’s area in the temporal cortex); the middle blue square represents the visual network underlying the visuospatial sketchpad and semantics, incorporating the dorsal (“where”) and the ventral (“what”) pathway; the right blue square represents the episodic buffer and short term memory, supported by activation of the hippocampus. Prefrontal cortex image from Wikimedia courtesy of Gray (1918): Brodmann areas 8 = primary motor cortex (eye fields), 9 = dorsolateral, 10 = frontopolar, 11 = orbitofrontal, 44/45 = inferior frontal (Broca’s area), 46 = dorsolateral, 47 = orbitofrontal. Broca’s/Wernicke’s area image from Wikimedia courtesy of Anatomy and Physiology, Connexions Web site. http://cnx.org/content/col11496/1.6/; Dorsal and ventral visual stream image from Wikimedia courtesy of Anatomy and Physiology, Connexions Web site; Hippocampus image from Wikimedia courtesy of Gray, 1918. **(B)** The original WM model by Baddeley and Hitch (1974), reproduced via Wikimedia.

In an attempt to highlight the mechanisms that may initiate impulse control, involving an interplay between bottom-up, non-conscious processes, and top-down, conscious cognitive processes in the healthy human brain, we turn to a neurocognitive description of the WM model by Baddeley and Hitch (1974) (**Figure 2**). In this model, a central executive (which is not in itself synonymous with consciousness or cognitive control, but rather an interplay between systems), residing within prefrontal cortex networks presides over what are termed *slave systems*. These slave systems, according to the model, are tripartite, interacting compartments known as the *phonological loop* (language), *visuospatial scratchpad* (visual semantics), and *the episodic buffer* (short term and episodic memory). These three WM subsystems and their neurobiological substrates will now be considered in more depth.

**FIGURE 3:**
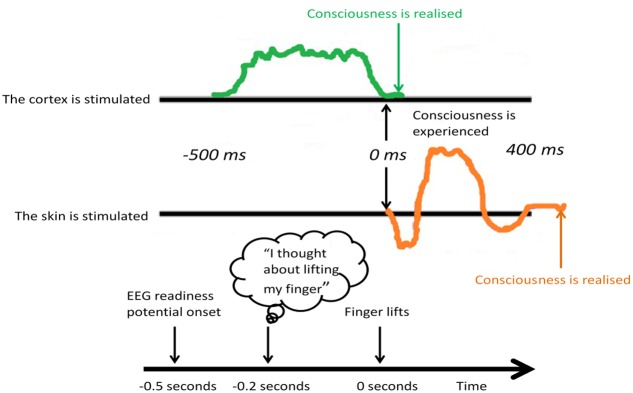
Libet’s half-second study. External stimulus of the cortex and internal experience. At least 500 ms was needed of cortical stimulation before subjective experience.

Firstly, the phonological loop, which may support cognitive ruminations, encompassing the articulatory loop and acoustic store, involves repetitive, conscious mental rehearsal strategies that promote the consolidation of beliefs (e.g., mantras), incorporating a dual network, namely speech production of Broca’s area in the frontal cortex (articulatory loop) and speech comprehension of Wernicke’s area in the temporal cortex (acoustic store). Secondly, interacting with the phonological loop are the dual visual semantic networks, namely the dorsal and ventral visual streams for action and perception (Goodale and Milner, 1992), or the “where” and the “what” pathway (Mishkin and Ungerleider, 1982), respectively. The dorsal “where” stream is primarily sensory, and follows a path from area V1 of the primary visual cortex (which can be activated by non-consciously processed stimuli, see Brooks et al., 2012c) to the parietal cortex, enabling a person to visualize the self-relevance of belief systems in time and space. Of note, it is traditionally the frontoparietal network that activates during WM functional magnetic resonance imaging (fMRI) studies (Rottschy et al., 2012). Conversely, the ventral “what” stream follows a path originating in V1 to the temporal auditory cortex (incorporating Wernicke’s area), with connections to the interoceptive (insular cortex) and episodic memory (hippocampal) regions, giving visual perception a comprehendible, concrete visual form in the mind. Thirdly, the episodic buffer can also be activated by non-conscious stimuli (Brooks et al., 2012c) and interacts with the phonological loop and visual semantic networks via the hippocampal-amygdala network residing close to Wernicke’s area in the medial temporal cortex. The hippocampal-amygdala network is integral to the mesolimbic reward/motivation pathway and interactions with prefrontal cortex, particularly in terms of building saliency and priority maps that influence overt and covert attentional systems (Zelinsky and Bisley, 2015). Poignantly, accumulating neurobiological evidence in animals and humans demonstrates direct prefrontal – hippocampal neural circuitry supporting the top-down modulation of bottom-up processes (Anderson et al., 2016). Furthermore, non-conscious processing of salient visual stimuli originating in V1 and the dopaminergic mesolimbic pathway, may occur up to half a second prior to conscious action tendencies (Libet, 1985), which may shed light on the sequence of activations of these sub-systems of WM in response to stimulation (**Figure 3**).

### Baars’ Global Workspace Theory (Baars et al., 2013)

Drawing on the Global Workspace Theory (Baars et al., 2013) can help to understand how the central executive of the WM model might preside over these slave systems in order to orchestrate conscious cognitive control of distracting stimuli. The central executive could also be related to the executive control network (ECN) that functions antagonistically to the default mode network (DMN). The ECN is usually related to externally focused goal-related cognitions, whereas the DMN reflects introspection, day-dreaming, and general self-monitoring. In brief, the Global Workspace Theory posits that the updating of conscious perception of fluctuating internal (related to DMN) and external (related to ECN) stimulation (e.g., under conditions of uncertainty) emerges from backstage non-conscious processing. In other words, the conscious stage accommodates, or receives signals from *transient* actors (of various sensory modalities, e.g., auditory, tactile, and visual) that are guided by a) priority maps and context setting about the self, world, and others (e.g., Zelinsky and Bisley, 2015; Trask et al., 2016) that may over time become unconscious, and (b) by an unconscious audience (self-precepts, automatisms, memories, and language). Recent functional connectivity analyses (e.g., hub detection and network-based statistics) have provided support for the Global Workspace Theory, demonstrating how functional networks in the brain reorganize according to higher cognitive loads, particularly during the *n*-back WM task (Finc et al., 2017). In particular, it appears that network modularity decreases as cognitive load (on the *n*-back WM task) increases; specifically, far-reaching network hubs increase but local hubs decrease in conjunction with greater global connectivity with the DMN (Finc et al., 2017). These most recent findings are pertinent in the consideration of the role of WM in cognitive control, suggesting the wider recruitment of backstage, non-conscious neural processes with increasing cognitive demand.

### Bayesian Probabilistic Inference or ‘The Bayesian Brain’

Progressing the Global Workspace Theory, Bayesian Probabilistic Inference, or the Bayesian brain (Nilsson, 1986; Friston, 2012) follows three main principles in terms of how the central executive of the WM system, driven by unconscious backstage processes, may guide decisions under conditions of uncertainty. Firstly, activation of the neural networks within the prefrontal cortex (e.g., anterior cingulate and medial prefrontal) and hippocampus (which supports non-conscious, episodic, and salient memories) likely conform to the *likelihood principle*. In other words, calculating prediction error and inferring (with generative models) the evaluation of current experiences (interoceptive and exteroceptive) with prior experience (“have I seen it before?”). Secondly, neural systems within the prefrontal cortex (e.g., dorsomedial and orbitofrontal), insular cortex, and basal ganglia (e.g., ventral striatum, hippocampus, and amygdala) process the saliency of stimuli to derive *frequentism* or in other words, familiarity, by merging prior experience with current beliefs about the stimulus (“did I like it before?”). Finally, *Bayesianism* confers belief systems, perhaps via activation of the dorsolateral prefrontal cortex (DLPFC), visuospatial and language networks, with frequency of exposure to a certain event in order to update predictions and action tendencies the presence of uncertainty (“how should I respond again now?”).

With the processes of the Bayesian brain and Global Workspace Theory combined, there occurs an *epistemic foraging* for information (sampling of intrinsic and extrinsic stimuli) until the best prediction about the uncertain future based on prior beliefs while reducing error and free energy, is found (Friston et al., 2015). Relating epistemic foraging to excessive cognitive control, as seen for example in AN, could reflect heightened ruminations, local versus global thinking and attention to detail (Kothari et al., 2013) that support the updating of prior, rigid cognitive models concerning eating, weight, shape, and food (Dell’Osso et al., 2016). Conversely, in studies measuring the ‘jumping to conclusions (JTC)’ bias – indicative of less epistemic foraging and reduced cognitive load – people with AN do not show such a bias (Wittorf et al., 2012; McKenna et al., 2014). Whereas, those with JTC bias have reduced epistemic foraging, collecting less information to arrive at a decision, which is linked to WM deficits and delusional thinking (Garety et al., 2013). As such, people with SUD, who are at risk of delusional and psychotic disorder, usually behave in a habitual, model free manner and ‘jump to conclusions’ (Wittorf et al., 2012; Voon et al., 2015). Considering the healthy brain for now, the transient stage of WM and the conscious illusion of cognitive control, presiding within PFC circuits and interacting with lower order circuits, is consciously experienced as a deliberative, generative inference (particularly if prediction error/free energy is high) that shapes predictions about future events/goals (Schwartenbeck et al., 2015). This is particularly perceived if cognitive load is high, forcing the brain to epistemically forage for a conclusion that solidifies or updates a prediction. Our brains measure the degree to which our predictions about uncertain events are in error, taking more measurements that are held in mind by delay interneurons (see below) for accurate updating of our prior beliefs. This dynamic updating is experienced consciously and is solidified in episodic memory (which ultimately becomes unconscious) to build our internal (sub)optimal cognitive models.

### Neural Processes of Working Memory in the Healthy Human Brain

Often studies of WM report insignificance in behavioral performance between healthy control groups and mental disorder populations, but when including brain imaging measures in a study, significant neural differences can often be found (Clark et al., 2017), and for review see below. This might suggest that the dynamic nature of the brain and neuroplasticity mechanisms support the development of compensatory processes that adapt to the unique experiences of the individual and which may, in turn, develop into entrenched, habitual mental disorder if not repeatedly challenged. Repetitive storage, encoding, and retrieval of processed stimuli (“have I seen it before, did I like it before, how should I respond again now?”) within the cortico-parietal-hippocampal circuitry underlies the detection of patterned activity and coincidental events (Basu and Siegelbaum, 2015). Such repetition underlies the dynamic mechanisms of Bayesianism as described above, and may be the process by which WM fosters neuroplasticity and alterations to the subjective experience of cognitive control. This innate neural learning mechanism could be harnessed to improve mental disorder (Lewis, 2015). As a starting point, basal ganglia processes may be at first non-consciously activated via the thalamus/brain stem, V1 and projections onto amygdala and striatal systems that register the saliency of a stimulus (“have I seen it before?”), particularly in terms of Pavlovian conditioning involving neocortex (Chau and Galvez, 2012; LeDoux, 2014). Such non-conscious saliency processes occur backstage, as *readiness potentials* – at least half a second before they decay (see discussions on meta-neuronal assemblies that register neuronal decay and the link to consciousness: Greenfield, 2017) and are registered by conscious systems in the higher order networks (Libet et al., 1979) (**Figure 3**). On this basis, subsequent “feeling” occurs (“did I like it before?”) through conscious cognitive processing of neural raw materials (LeDoux, 2014).

The interface between backstage processes and conscious on-stage processes, in other words cognitive–affective interaction, likely begins between the basal ganglia (e.g., amygdala, hippocampus, and striatum), anterior cingulate cortex (ACC) and via direct connectivity to the DLPFC for prediction error detection, reinforcement learning, and Bayesian updating about how to respond (Garrison et al., 2013). The ACC, which is involved in the prediction error detection network (Shen et al., 2015) contributes to the larger mesolimbic pathway, of which the saliency network of the amygdala-hippocampal-insula subsystem is a part and can be activated by non-conscious stimuli (Brooks et al., 2012c; Meneguzzo et al., 2014). Furthermore, it is intriguing to consider that non-conscious episodic memories via the slave systems (e.g., amygdala-hippocampal) can be triggered by stimuli that have been frequently encountered and are therefore salient and specific to the individual, underlying the qualitative nature of adaptive behavior (Desmedt et al., 2015).

**FIGURE 4:**
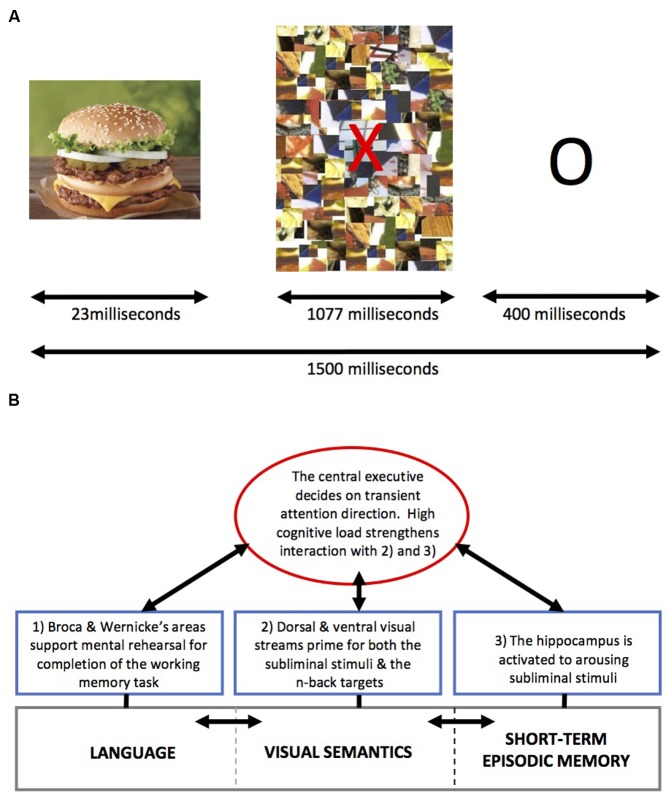
**(A)** Schematic diagram of the backward masking technique. A salient image (e.g., food) is presented for 23 ms, prior to a mosaic screen. The mosaic functions as a backward mask to interrupt the processing of the salient stimulus rendering it subliminal. Adorned on the mosaic image is a red letter, which changes during consecutive cycles, and represents either a target or non-target image during the *N*-back task. For 1-back, the target letter is the same as the previously presented letter, whereas for 2-back the target letter is the same as the letter presented two letters previously. Image courtesy of Dr. Samantha Brooks’ Ph.D. thesis (Published via University of London, King’s College, London, June 2010). **(B)** Using a schematic diagram of the classic WM model by Baddeley and Hitch (1974), reproduced via Wikimedia, to hypothesize as to the sequence of events that is associated with competitive interference during subliminal priming when cognitive load is low, and reduced competitive interference when cognitive load is high in healthy subjects (see Uher et al., 2014). (1) During the subliminal presentation of arousing stimuli (e.g., food and aversive image), which have been frequently encountered before (e.g., food) or that evolutionarily we are primed to find salient (e.g., aversive image of bloody bodies), the hippocampus is activated after the visual stimulus, via V1 and thalamus has increased dopamine release in the mesolimbic pathway. Direct connection to the prefrontal cortex from the hippocampus ensures that cognitive systems are primed to attend to this stimulus for further processing.

The interference effects of non-conscious *backstage* salient processes on WM in healthy adult participants has been examined by manipulating the presentation of different types of subliminal images (appetitive, aversive, and neutral) while varying the levels of cognitive load (Uher et al., 2014). The rationale for the study was to probe how cognition and emotion interact, in terms of modulating affective arousal and cognitive engagement, since it is proposed by various groups that emotion and cognition compete for limited resources in the brain (Desimone and Duncan, 1995; Lavie, 2000; Pashler et al., 2001; Pessoa, 2009), particularly in the prefrontal-parietal cortex (Jaeggi et al., 2003; Rottschy et al., 2012). Against this background, in this study we showed a scrambled mosaic image on screen that functioned as a *backward mask*, commonly used in subliminal studies (**Figure 4**). The backward mask was presented directly after a 20-ms presentation of an aversive (e.g., bloody bodies), appetitive (e.g., high calorie food), or neutral (e.g., utensils) image from the International Affective Picture System (IAPS) (Lang et al., 1996) that were previously rated by independent volunteers in terms of pleasantness, aversion, salience, visual complexity, and recognizably. During the presentation of these subliminal stimuli to a group of healthy adult men and women (mean age: 25 years), we engaged participants in the easier 1-back and more difficult 2-back versions of the *N*-back WM task (Kirchner, 1958). In conjunction with our hypotheses, we found that when WM load was low, competitive interference between cognitive (e.g., completing the task) and affective processes (e.g., neural responses to subliminal arousing stimuli) on prefrontal cortex attentional systems was high (e.g., there was an increase in errors). However, increasing the cognitive load of the WM task appeared to attenuate the interfering effects of subliminal arousing stimuli (of both positive and negative valence).

Our data complemented findings by others showing that increasing the cognitive load can reduce the interference caused by consciously processed emotional distracters (Van Dillen and Koole, 2009; Van Dillen and Derks, 2012). Our findings were particularly interesting given that a post-experiment forced choice test confirmed that all participants were unaware of the subliminal interfering images during the study. From this we suggested that affective interference does not directly relate to conscious evaluation of the current cognitive activity (e.g., *metacognition*), and that a top-down regulatory mechanism within the prefrontal cortex appears to exist that is not reliant on conscious metacognition. Thus, it might be that hippocampal mechanisms in response to subliminal stimuli are activated first by the dopaminergic mesolimbic pathway via area V1, such that WM processes (visual semantics and language) are distracted if cognitive load is low (1-back). However, when cognitive load is high (2-back), the central executive might bias visual semantic and language systems in favor of prior goals (e.g., completing the task) such that hippocampal and salient environmental activation does not impinge on WM processes. For a schematic illustration, see **Figure 4**.

In line with our findings above (Uher et al., 2014), the central executive component of the WM model, particularly involving DLPFC-ACC-insula coupling (Fang et al., 2016) may regulate non-conscious stimulation of the hippocampus to reduce distraction when cognitive engagement is high (e.g., excessive epistemic foraging). Specifically, a rostral (bordering the evaluative OFC) versus caudal (bordering the error detecting ACC) involvement of the DLPFC may ensue according to low versus high cognitive load, respectively (Rottschy et al., 2012), and may be a robust model of cognitive control to consider for excessive cognitive control in AN. Similarly, this may support the notion of WM training to strengthen a person’s ability to evoke WM at a higher load/capacity in order to prevent distraction. However, interactions between lower order non-conscious slave systems and higher order ECNs are also mediated by genetic and epigenetic factors that underlie traits and differing thresholds of cognitive control and WM capacity that we consider next.

### Genetic Mechanisms Underlying the Working Memory Process

In terms of the genetic influences on WM processes, particularly concerning neuroplasticity, it is useful to consider expression within some of the main cellular signaling pathways that are implicated, including dopaminergic (D1–D5 receptors, DAT, and COMT), glutamatergic (BDNF and NMDA) and GABAergic (inhibitory interneuron) systems (Karlsgodt et al., 2011).

#### Dopaminergic

The link between dopamine and WM, particularly involving the DLPFC and related neurocircuitry and the ability to generate and hold in mind visual representations in the absence of external stimulation, has long been established (e.g., Goldman-Rakic, 1998). Expression of D1 receptors are predominantly observed in the prefrontal cortex, and D2/D3 receptors in the striatum (Charuchinda et al., 1987; Lidow et al., 1991). The link between expression of D1 receptors and WM performance follows an inverted U-shape, whereby low levels of D1 receptors in the prefrontal cortex are associated with aging, neurodegenerative disease (e.g., Parkinson’s and Alzheimer’s) and poorer WM performance, while high levels of D1 receptors are associated with impulsivity, stress, psychosis and impaired WM function (Abi-Dargham, 2003; Vijayraghavan et al., 2016). D2/D3 receptors by contrast are predominantly found in the basal ganglia and have been linked to episodic memory and also drug craving (Morales et al., 2015; Nyberg et al., 2016), and upregulation of D2 receptors in the basal ganglia is observed following WM training in young adults (Söderqvist et al., 2014). Furthermore, activation of D4 receptors in the medial prefrontal cortex is linked to biasing of decision-making under aversive conditions (Floresco and Magyar, 2006). Finally, reduced D5 receptor levels in the prefrontal cortex are associated with downregulation of NMDA receptors in the hippocampus and diminished long-term memory, but not specifically WM performance (Moraga-Amaro et al., 2016), suggesting that the D5 dopamine receptor system may contribute to Bayesian updating and consigning information from epistemic foraging to unconscious memory, which might be considered an important part of the loop of repetitive WM functioning.

The role of single nucleotide polymorphisms (SNP) on the catechol-*O*-methyltransferase (COMT) gene in WM function is well documented and may be associated with the inverted U-shape relationship between dopamine levels and WM function mentioned above (Schacht, 2016). COMT is an enzyme (or catabolite) that degrades dopamine, rendering it inactive, and is particularly important in regions that have low presynaptic dopamine transporter (DAT) expression, such as the prefrontal cortex (Matsumoto et al., 2003). Specifically, COMT val158met SNP (rs4680) confers variation in COMT efficacy and dopamine tone, especially in relation to D2 regulation of receptors downstream in the basal ganglia. Individuals homozygous for the valine COMT allele, which more rapidly degrades dopamine, have significantly lower prefrontal cortical dopamine, which is linked to impulsivity, whereas those homozygous for the methionine allele display a slower degradation of dopamine, which is linked to higher levels of cortical dopamine and schizophrenia (Schacht, 2016). Thus, optimal degradation of dopamine within the prefrontal cortex, following optimal release of dopamine from basal ganglia likely supports effective WM processing.

#### Glutamatergic

The genetic expression of brain derived neurotrophic factor (BDNF) is linked to glutamatergic pathways and plays a crucial role in neuroplasticity by mediating changes in cortical thickness and synaptic density, particularly within the corticolimbic pathway, with higher serum levels of BDNF associated with better WM (Håkansson et al., 2016). Moreover, the synergistic interactions between neuronal activity and synaptic plasticity (and receptor upregulation) by BDNF underline it as an essential regulator of cellular processes for WM, particularly hippocampal long-term potentiation (Lu et al., 2014). Similarly, there is strong evidence that BDNF is a good candidate for experience-dependent modulation of neural systems underlying learning and memory (Korol et al., 2013), in line with the Bayesian brain process described above. In line with this, a study examined 367 healthy elderly Swedish men and women for the BDNF functional rs6265 SNP, WM and brain volume, and found that the Met allele was associated with better WM performance and larger cerebellar, precuneus, left superior frontal gyrus and bilateral hippocampal volume, and smaller brainstem and bilateral posterior cingulate volume (Brooks et al., 2014a). WM ability, and the efficacy of repetitive training to evoke neuroplasticity changes, particularly within the basal ganglia networks, may be significantly associated with BDNF expression, such as upregulation of dopaminergic mesolimbic receptors and subsequently reduced levels of dopamine arriving at the prefrontal cortex from the basal ganglia. This may in turn be reflected in volumetric changes in the basal ganglia and improved levels of impulsivity in those with SUD (Brooks et al., 2016).

The *N*-methyl-d-aspartate receptor (NMDAR) is glutamatergic and expressed in relation to the gene dystrobrevin binding protein-1 (dysbindin, or DTNBP1) in layer III-V excitatory pyramidal cell circuits of the DLPFC that are of primary importance when considering the genetic influences on WM function (Karlsgodt et al., 2011; Arnsten and Wang, 2016). Specifically, dysbindin contributes to delay interneurons found in DLPFC layers that continue to fire across time in the absence of direct stimulation, which are excited via NMDARs (Wang et al., 2013) and may contribute to WM capacity. Moreover, spatial tuning of delay cells within the DLPFC is cultivated via lateral inhibition from GABA interneurons (Goldman-Rakic, 1995), which differs to other classical circuits, for example those found in occipital cortex V1 that predominantly activate feed-forward as opposed to lateral inhibition (Gabernet et al., 2005).

#### Gamma-amino Butyric Acid (GABA)

Antagonistically to the excitation of NMDAR via glutamatergic pathways, effective WM function additionally involves activation of inhibitory GABA interneurons (Goldman-Rakic, 1995). It is suggested that a discrete balance between excitatory NMDA and inhibitory GABAergic neurons in the PFC allows for the specificity of the WM trace during repetitive cognitive tasks (Garavan et al., 2000), and that disruption to oscillatory activity in the gamma-band range can jeopardize WM performance (Lewis and Moghaddam, 2006). Thus, against this background, gene expression, particularly involving layer III-V pyramidal cells within the prefrontal cortex that rapidly and reversibly alter the strength of synaptic connections underlying dynamic network connectivity likely contribute to repetitive Bayesian dynamic updating and improvements to WM (Arnsten and Wang, 2016).

#### Epigenetic Effects

Alongside genetic linkage studies into WM function, accumulating evidence highlights the link between repetitive environmental stimulation, as well as epistemic foraging (particularly when exposed to significant stressors and during memory formation) and epigenetic effects, which are defined as heritable changes in gene transcription and/or phenotypic alterations that do not follow changes in DNA sequences (Jablonka, 2012). Most commonly studied epigenetic effects are modifications of four histones (H2a, H2b, H3, and H4) present in chromatin (Gelato and Fischle, 2008) and DNA methylation (Smith and Meissner, 2013) (for extensive review of these two processes, see Cadet, 2016). The epigenetic effects of WM are of fundamental importance in terms of how the mind can change in response to unique environment stimulation (e.g., repetitive WM usage and/or training) and in turn, how these changes impact future interactions with the environment (for extensive review of neuroepigenetics of memory, see Mikaelsson and Miller, 2011). For example, memory storage, consolidation and retrieval require long-term increases in synaptic strength that are supported by transcription and chromatin modification and that underlie neuroplasticity (Mikaelsson and Miller, 2011). The first animal studies of the epigenetic effects of memory formation (for taste preference) demonstrated chromatin-related changes in the insular cortex (Swank and Sweatt, 2001) and the hippocampus (for fear learning) (Levenson et al., 2004).

Specific epigenetic effects of early life deprivation (social isolation) on WM-related brain structures in rats has been shown to increase H3 acetylation at the BDNF gene and BDNF protein expression, specifically in the medial prefrontal cortex, and decreased BDNF mRNA levels, H3 acetylation of the BDNF gene and BDNF protein expression in the hippocampus (Li et al., 2016). Similarly, the BDNF SNP rs6265 (Val (66) Met), which creates or abolishes a CpG dinucleotide for DNA methylation, is associated with modulation of prefrontal cortex activation, impaired WM accuracy and increased methylation in ValVal subjects, but improved WM and reduced methylation in ValMet subjects (Ursini et al., 2016). Epigenetic effects in terms of DNA methylation in the MB-COMT promoter gene, which is associated with WM function, have recently been found to correlate with left DLPFC activation during the performance of a WM task in humans (Walton et al., 2014). Furthermore, early life deprivation has been shown to negatively affect cortical GABA function in adult life, including impaired prefrontal expression of enzymes required for GABA synthesis (Labouesse et al., 2015), which likely has an adverse effect on WM. Epigenetic effects are associated with WM as summarized above, and play a major role in the development and maintenance of psychiatric disorder (Karsli-Ceppioglu, 2016; Luoni and Riva, 2016).

Having now summarized theories of WM, as well as some of the neuropsychological, neural and genetic processes underlying WM in the healthy human brain, we now systematically review studies of WM in AN and SUD – psychiatric populations considered to be at opposing ends of an impulse control spectrum (**Figure 1**) (Brooks et al., 2012b; Brooks, 2016).

## Anorexia Nervosa, Working Memory, and Cognitive Control

### Neuropsychological and Clinical Considerations

The current systematic review has found that to date 16 studies (published between 2010 and 2017) have examined WM performance in AN. In a previous review of all studies examining WM to date (the first study in 2002), it was found that 45% of studies showed patients with AN to have better WM performance than healthy subjects or those who binge; 18% reported worse performance and 37% reported no difference (Brooks, 2016). While the majority of studies in the previous review report superior performance, the heterogeneity of findings is likely due to the mixing of eating disorder subtypes, differing durations of illness and experiments that test different components of WM (e.g., verbal versus visuospatial). For example, varying degrees of restriction versus binge eating concomitant with switching between subtypes of ED over the course of illness can influence WM ability, with those with AN sometimes having better, but also worse WM ability (e.g., Israel et al., 2015; Weider et al., 2015). A longer duration of illness can also influence WM function (Dickson et al., 2008; Pruis et al., 2012; Lao-Kaim et al., 2014), but often WM ability does not correlate with clinical eating disorder measures (e.g., Seed et al., 2002; Fowler et al., 2006; Hatch et al., 2010; Nikendei et al., 2011), suggesting that clinical symptoms during chronic eating disorder are likely transient and secondary to the core cognitive disturbances in those who develop ED that often manifest during adolescence (Lena et al., 2004).

Since the last review in 2016, an additional six studies have been found that examined WM in AN (Phillipou et al., 2015; Biezonski et al., 2016; Giombini et al., 2016; Bentz et al., 2017; Ritschel et al., 2017; Solstrand Dahlberg et al., 2017). Of these six studies, none reported better WM performance in AN compared to HC; some reported normalization of WM function after treatment such as cognitive remediation therapy (CRT) and in-patient treatment (Castro-Fornieles et al., 2010; Giombini et al., 2016), whereas others show that abnormal function remains on recovery (Ritschel et al., 2017). This leaning toward the reporting of worse WM performance in AN may reflect the fact that there may be heightened WM capacity in AN but that this is utilized primarily for the exercising of detailed, rigid, complex cognitions about eating-related concerns. Furthermore, contemporary studies examining AN and WM now seem more often to combine neuropsychological measures of WM with neural measures using brain imaging techniques, which may shed better light on the mechanisms involved in cognitive control. For example, Biezonski et al. (2016) demonstrate thalamo-cortical structural and functional abnormalities in those with AN coincide with deficits on tasks probing WM and cognitive control. Similarly, increased temporoparietal activation in those who have recovered from AN, during a probabilistic learning task suggests a trait marker for excessive cognitive control and inefficiency to learn from unfolding new experience (Ritschel et al., 2017). These findings are suggestive of rigid, increasingly detailed prior beliefs (related to eating and body image) that are supported by excessive WM function that are difficult to alter (e.g., with cognitive therapies alone). Furthermore, larger insular cortex volumes in adolescent females with a new diagnosis of AN are related to significantly slower WM reaction time (Solstrand Dahlberg et al., 2017) and may be reflect proneness of hypersensitivity to distraction (Dickson et al., 2008). Taken together, the recent studies suggest a suboptimal WM performance in those with AN that coincides with hyperactivation of brain networks associated with excessive cognitive control and the perception of saliency (e.g., thalamo-cortical, temporal, parietal and insula). This may support a Bayesian brain notion that those with AN engage in excessive epistemic foraging of intrinsic and extrinsic stimuli to strengthen proof for generative models of future, uncertain goals (pertaining to concerns about eating, shape, weight, and food – “I will only eat a salad at noon”). Such excessive, chronic epistemic foraging may foster neural changes that support habitual, rigid, attention-to-detail style thinking that maintains AN, and that influencing cognitive changes at the neural level (e.g., with WM training) may improve treatment efficacy for AN. It is noteworthy that no studies have yet, to the authors’ knowledge, examined the (epi)genetic underpinnings of WM function and neuroplasticity in AN.

Other research into eating disorders supports the notion of Bayesian brain processes in the development of AN. For example, a diagnosis of restricting AN is linked to perceived self-control and delayed gratification in contrast to binge eaters, supporting a transdiagnostic, spectrum model of impulse/appetite control (Bartholdy et al., 2017). Furthermore, to support the spectrum view of impulse control in eating disorders, AN appears to be protective for the development of SUD (Kaye et al., 2013b). Alongside more prominent clinical symptoms (Treasure and Schmidt, 2013), WM is linked to core deficits, for example that pertain to excessive appetite control by way of repetitive cognitive ruminations. In support of this, a link has recently been demonstrated between AN, negative affect and disease-related ruminations (Seidel et al., 2016). This finding is in line with recent work on the Bayesian brain by Friston et al. (2015; Peters et al., 2017) suggesting that high levels of uncertainty in the environment regarding future events (e.g., high prediction error, free energy/entropy within brain processes) may lead to excessive epistemic foraging and stress. This can be related to negative affect/allostatic overload and to the findings of Seidel et al. (2016), in that if people with AN are excessively ruminating on future goals and attempts to control eating, body shape and weight, they place themselves in a cognitively uncertain situation. This is because, in a world where food and body images are abundant, there is uncertainty about whether appetite suppression and control of the body can indeed be achieved over the longer term. The tension between uncertainty versus excessive epistemic foraging to support prior beliefs would increase allostatic load and result in heightened stress/anxiety, which is commonly comorbid in those with AN. However, it may also be rewarding to experience achievement of appetite control goals in the face of uncertainty/abundance of food/eating/body image cues (O’Hara et al., 2015).

Similarly, it has been demonstrated that an early diagnosis of eating disorder during adolescence is linked to obsessive-compulsivity regarding concerns about shape, weight, and eating (Brooks et al., 2014b). Furthermore, the study also reported that higher obsessive-compulsive scores correlated with slower WM performance on the *N*-back task. Slower WM performance may be indicative of an interference effect, for example, if the brain is already engaged in ruminations about shape, weight, and eating (triggered non-consciously by images of food). That WM is distracted by affective stimuli in those with AN compared to healthy, and more so as duration of illness progresses, has been corroborated by Dickson et al. (2008). Similarly, another study found that WM performance in adult females with AN is only comprised by non-consciously processed images of food, not other affective subliminal images (Brooks et al., 2012a). Also, the interference effect observed in this study only occurred when neural systems were engaged in a WM task that utilized DLPFC-related processes, and not a conflict monitoring task that utilized ACC-related networks (Brooks et al., 2012a). Taken together, research suggests that WM function may be a feature of excessive rumination and appetitive control in those with AN, and that as the disorder progresses, visual and phonological episodic memories (e.g., images of food and body) become rigidly ingrained in the non-conscious saliency network due to their frequent and repetitive processing, which ultimately influences neuroplasticity processes and renders DLPFC-related neurocircuitry function suboptimal (e.g., biased toward detailed eating disorder cognitions).

### Neural Processes of WM in AN

Rumination and appetitive control in adolescents and adults at risk for developing AN may at first be deliberative and recreational (e.g., occasional dieting to achieve a visualized weight/body-image goal), but may switch to become ingrained, compulsive and habitual, akin to the neurobiological process of addiction (Everitt, 2014; O’Hara et al., 2015). For example, WM processes rely on the dopaminergic corticolimbic pathway (Goldman-Rakic, 1998), and as such, repetition of phonological and visual images may activate the saliency network, which over time may lead to epigenetic changes that upregulate dopamine receptors in line with altered dopamine levels in the mesolimbic pathway. In neural circuitry terms, deliberative responses appear to activate prefrontal cortex and ventral striatal (nucleus accumbens) networks linked to incentive salience and decisions about wanting and liking, whereas compulsive and habitual responses switch to dorsal striatum (Everitt, 2014; Koob and Volkow, 2016). Repetitive ruminations in those with AN (e.g., “I will only eat salad at noon”), from a WM model perspective, may engender the episodic representation of images evoked by deliberative prefrontal cortex predictive processes, such that internally generated images are eventually furnished with a saliency akin to a concrete object.

Additionally, AN is linked to hypoactivation in the insular cortex – a saliency hub – in response to food images, which is linked to reduced interoception, whereas by comparison the exercising of restraint cognitions to be thin appears more rewarding and addictive (e.g., “*nothing tastes as good as skinny feels*,” Kaye et al., 2013a). Epigenetic effects in dopaminergic receptor populations within the related basal ganglia network eventually leads to withdrawal, stress, and anxiety (Koob and Volkow, 2016) as more drug consumption – or in the case of AN, restraint cognitions of increasing complexity and detail – need to be exercised, as habituation ensues (Kaye et al., 2015). However, the degree to which WM is exercised according to restraint cognitions is likely transient, determined by current ED subtype and may be translated into neurobiological and epigenetic effects underlying the strength and efficiency of neural networks supporting WM capacity. Considering the potential neurobiological underpinnings of cognitive control and WM in AN, one study observed that while the global brain volume was smaller, the right DLPFC volume negatively correlated with age (between 18 and 45 years) in healthy adults but not in adults with a chronic diagnosis of restricting AN (Brooks et al., 2011a), suggesting that some difference in functionality might save the AN DLPFC from age-related normal atrophy. With this in mind, when weighting for BMI, the right DLPFC volume in RAN positively correlated with restraint cognitions (as measured by the eating disorder examination questionnaire) (Brooks et al., 2011a). While the study was a small pilot, it is intriguing to consider that excessive ruminations over a substantial time period (e.g., chronic eating disorder over approximately 30 years lifespan), measured cross-sectionally in individuals with differing durations of illness, may alter neural plasticity and therefore DLPFC volume.

In this vein, numerous recent studies have linked increased DLPFC function and related regions to WM and disease-related processes in those with AN. For example, a study revealed that increased neural activation during the perception of high calorie visual food stimuli occurs in the bilateral prefrontal cortex (including the DLPFC and ACC) in adolescents with an early diagnosis of eating disorder, as well as adults with chronic AN compared to patients with bulimia nervosa (Brooks et al., 2011c), and that increased DLPFC activation positively correlates with high obsessive-compulsive scores and slower WM performance (Brooks et al., 2014b). Other groups have also linked WM ability in those with restricting AN compared to healthy controls, to greater right DLPFC activation as cognitive load increases (Israel et al., 2015). Moreover, others have suggested that neural activation differences during WM performance in the DLPFC between patients with AN and controls may be distinguished by duration of illness (Lao-Kaim et al., 2014). Also, in a recent review of resting state functional MRI studies of AN, connectivity deficits in corticolimbic networks pertaining to cognitive control were implied (Gaudio et al., 2016). Similarly, a recent study by Boehm et al. (2016) demonstrates that passive (e.g., not engaged in a perception or cognitive task during the scan) functional resting state connectivity was reduced between the DLPFC and frontoparietal network in people who had recovered from AN, suggesting that aberrant WM and cognitive control is a trait marker for the risk of developing an ED. Similarly, increased DLPFC activation while perceiving and responding to images of food (e.g., “how disgusting do you find the images?”) in recovered patients who previously had chronic AN, may not be an indication of ‘good outcome’ (Uher et al., 2003), but rather, a reflection that underlying core cognitive symptoms of aberrant WM are recalcitrant. Taken together, these data suggest that the disruptive influence of non-conscious processes on WM and related attentional bias (Brooks et al., 2011b) increases as AN progresses, which in turn promotes epigenetic changes within the cognitive-affective neural interplay underlying the cognitive control of appetite.

### Genetic Influences on WM in AN

Changes in DLPFC volume as measured by MRI for example, may translate into epigenetic effects and neuroplasticity within prefrontal cortex networks that support WM function. In line with this suggestion, increased volume in the DLPFC may reflect similar findings of increased BDNF SNP rs6265 methylation in ValVal subjects and improved WM (Ursini et al., 2016). Increased volume in the prefrontal cortex could also reflect epigenetic effects within the COMT promoter gene, which is associated with WM function and modulation of prefrontal cortex during WM in humans (Walton et al., 2014). Similarly, prefrontal cortex expression of enzymes required for GABA synthesis and the function of delay interneurons may also be associated with differential structure and function in the DLPFC (Labouesse et al., 2015). However, according to the authors’ knowledge, no studies have yet examined the genetic underpinnings of WM processes in AN and the link to excessive cognitive control.

Against the background of the neurobiology of WM mechanisms associated with AN, the genetic epidemiology of eating disorders has recently been reviewed (Bulik et al., 2016). Firstly, the review highlights that all eating disorders appear to be heritable conditions that are determined by genetic and environmental interaction, which is pertinent to consider here, given that repetitive ruminations about food restriction and environmental stimuli perceived to be relevant (e.g., one’s own shape, weight and eating in comparison to others) may alter cellular brain processes. In this vein, while Bulik et al. (2016) admit that as yet, *genome-wide association studies (GWAS)* for eating disorders are still underpowered, the top hit (co-morbid with bipolar disorder) for AN is on the SOX2-OT gene on chromosome 3 (Boraska et al., 2014; Liu et al., 2016). The SOX2-OT gene is associated with neurogenesis, and might underlie varying degrees of neuroplasticity and the extent to which neural circuits are rigidly set or can be modified; however, this suggestion is currently speculative. Secondly, the review underlines that a novel method of analysis, namely linkage disequilibrium score regression (LDSR) has demonstrated a strong positive genetic correlation between AN, schizophrenia and bipolar disorder, which suggests that the common variants cumulatively associated with schizophrenia and bipolar risk also increase risk for AN (Bulik-Sullivan et al., 2015) – implicating dopaminergic processes that are associated with WM. Given the strong links between WM and cortical plasticity in those with schizophrenia (Genevsky et al., 2010), the genetic linkage between schizophrenia and AN further implicates WM in pathology.

Another recent review has considered not only the genetic epidemiology of eating disorders but also specific epigenetic links (Yilmaz et al., 2015), which link neuroplasticity and WM processes. Firstly, while anxiety and related disorders (e.g., obsessive-compulsive disorder) are highly comorbid with eating disorders and as such genetic analyses of genes related to serotonergic systems have been extensively studied, Yilmaz et al. (2015) report mixed and underpowered findings. Similarly, while there is some indication that dopamine receptor studies are implicated in levels of binge eating and attention deficit disorder in those with eating disorders, Yilmaz et al. (2015) suggest that replication studies are needed. Furthermore, while leptin receptor and melanocortin genes are associated with weight regulation, Yilmaz et al. (2015) report no significant and consistent linkage to those with eating disorders.

Yilmaz et al. (2015) hint that it is perhaps BDNF that holds the most promise as an epigenetic candidate for eating disorders, given that BDNF is involved in appetite suppression by regulating melanocortin signaling in the hypothalamus (Xu et al., 2003), although no strong links are currently found. Yet, it is interesting to consider, given that the BDNF SNP rs6265, which creates or abolishes a CpG dinucleotide for methylation, is associated with modulation of prefrontal cortex activation in terms of improved WM and reduced methylation in ValMet subjects (Ursini et al., 2016). Thus, while differences in nucleotide sequence between AN and controls may not differ significantly, it is possible that gene expression or methylation patterns, perhaps in BDNF SNP rs6265, may significantly vary in those with eating disorders in relation to measures of WM capacity. Yilmaz et al. (2015) report that due to the link with reward processes, the dopaminergic system has been most extensively studied in terms of epigenetics in eating disorders, which is also pertinent given the role of dopamine in WM (Goldman-Rakic, 1998). Accordingly, there is some indication that those with AN may have increased DAT (SLC6A3; also referred to as DAT) mRNA expression due to hypermethylation of the gene’s promoter region, as well as DRD2 promoter hypermethylation (Frieling et al., 2010), although this is yet to be replicated. And at the time of writing, there has been no convincing links between COMT epigenetic effects and eating disorders. Taken together, given their influence on corticolimbic circuitry, it might be that BDNF and dopaminergic epigenetic mechanisms are most pertinent to the examination of fluctuating WM ability and the link to cognitive control of appetite in those with AN.

## Substance Use Disorders Working Memory and Cognitive Control

### Neuropsychological and Clinical Considerations

At the opposite extreme of the impulse control spectrum model (**Figure 1**) are those who have reduced impulse control (e.g., binge eaters/SUD, Volkow and Baler, 2015) as proposed by the dual process theory of addiction (Bechara, 2005). With SUD (including stimulant, nicotine, opioid, marijuana, and alcohol use) in mind, we conducted a second systematic review yielding *n* = 93 studies that have examined WM and cognitive control, between 2010 (e.g., the start of this decade) to the present (August 2017) (Supplementary Table S2). The majority of studies, *n* = 68 (72%) reported worse WM performance compared to healthy drug-naïve controls or non-drug taking control groups, but it is not clear whether WM deficits are a trait (e.g., cause) or a state (e.g., consequence) of SUD. In attempt to probe the trait versus state effects of SUD, researchers have examined whether WM deficits normalize following a period of abstinence, and in some cases, there is moderate reversal of cognitive deficits following abstinence (Vonmoos et al., 2014). Similarly, in a recent randomized control trial, Bell et al. (2017) reported that abstinence during 3 months of CRT and work therapy for outpatients with SUD was related to improved WM function also present at 6 months follow-up. Furthermore, 3 weeks of marijuana abstinence led to WM improvements in adolescents between the ages of 15–19 years, but attention deficits remained (Hanson et al., 2010). However, withdrawal from heavy substance use (alcohol and drugs) during adolescence may lead to long-lasting, WM deficits that are related to neurotoxicity in later life (Hanson et al., 2011). Nevertheless, across the board, the resounding conclusion from these studies is that WM in SUD is deficient, but that there is some indication that deficits may not be long-lasting and can be improved.

At the opposite end of an impulse-control spectrum, where AN represents excessive cognitive control, conversely those with addiction behaviors, and SUD in particular, represent low cognitive control and impaired self-regulation (Bechara, 2005; Bickel et al., 2011, 2015; Volkow and Baler, 2015; Brooks, 2016; Squeglia and Cservenka, 2017). Given that dopaminergic systems underlying WM (Goldman-Rakic, 1995, 1998) are implicated in addiction (Volkow and Baler, 2015), WM deficits are considered to contribute to core pathology of addiction (e.g., Bechara, 2005). In support of this (but also in support of trait deficits) adolescents with a family history of alcoholism have been shown to be slower on a verbal WM task than those with no family history of alcoholism (Cservenka et al., 2012). And generally, those with alcohol use disorder are usually impaired on the WM executive function domain (Chanraud et al., 2010; Bogg et al., 2012), particularly in a Bayesian brain sense when attempting to make decisions under uncertain conditions (Brevers et al., 2014). Verbal WM is associated with the phonological loop (articulatory loop and acoustic store; Broca and Wernicke’s area, respectively), and so deficits in this – as opposed to the visuospatial – domain may prevent the effective verbal rehearsal of top-down cognitive strategies and recall of future goals (that may underlie excessive ruminations in those with AN, for e.g.). Rehearsing cognitive strategies verbally in mind might be even more difficult for those with SUD in the presence of distractors (McClure and Bickel, 2014). In line with deficits in rehearsing verbal strategies in mind, in those with impulse control disorders, various studies have found that attention deficit symptoms (e.g., impulsivity, “behavioral under-control,” fidgeting) and dysfunctional affective regulation during childhood predict the later development of SUD (Block et al., 1988; Caspi et al., 1996; Màsse and Tremblay, 1997; Moffitt et al., 2011). Moreover, in children with a diagnosis of ADHD there is a higher risk for developing SUD later in life (Molina and Pelham, 2003).

A formal diagnosis of ADHD has been shown to be dissociable in core WM brain regions in adult ADHD patients with and without spatial WM deficits (Mattfeld et al., 2015), suggesting that ADHD symptoms are somewhat necessary but not sufficient to reduce WM ability. This could be due, in part, to the development of compensatory neural mechanisms during adulthood that reduce WM deficits, or that core WM deficits in those with ADHD at risk for SUD aggregate on neural networks underlying the verbal and not spatial WM domain. With this in mind, a recent meta-analysis of adults with SUD showed significant deficits in the verbal WM domain and not others (Baldacchino et al., 2012), which may also reflect evidence that there is a switch from controlled (associated with ventral striatum and prefrontal cortex) to habitual (associated with dorsal striatum and amygdala) drug taking as SUD progresses (Everitt, 2014). In other words, recreational and controlled drug use may be associated with the employment of verbal WM strategies that help to curtail impulsive behaviors, which may be lessened during the switch to habitual drug use and withdrawal. This switch could also hint at epigenetic effects, combined with the evidence that improvements to WM and prefrontal cortex activation have been observed in substance users who have achieved a period of abstinence (Schulte et al., 2014).

### Neural Processes of WM in SUD

Further clues regarding the link between SUD and deficits in WM/cognitive control can be derived from the neuroimaging studies found in our systematic review (*n* = 31) employing electroencephalography (EEG), diffusion tensor imaging (DTI), single photon emission computed tomography (SPECT), functional and structural magnetic resonance imaging (MRI), including task – and resting state fMRI. Eighteen (58%) of these neuroimaging studies report normal WM performance but with altered, perhaps compensatory – or inefficient – neural processing (Chanraud et al., 2010, 2013; Crego et al., 2010; Jager et al., 2010; Schweinsburg et al., 2010; Sweet et al., 2010; Vollstädt-Klein et al., 2010; Bustamante et al., 2011; Sutherland et al., 2011; Bach et al., 2012; Bogg et al., 2012; Campanella et al., 2013; Charlet et al., 2014; Cousijn et al., 2014a,b; Ma et al., 2014; Loughead et al., 2015; Brooks et al., 2016). Neural and not behavioral differences during WM task performance may relate to the Global Workspace Theory and the recruitment of neuronal networks that activate the DMN (e.g., for self-reflection) when cognitive load is high (Finc et al., 2017). To support the notion that brain imaging data can provide additional insight into the processes of WM than behavioral data alone, a recent study has suggested that compensatory neural mechanisms are at play during variation in cognitive load (Clark et al., 2017).

The neuroimaging studies of WM in SUD have reported that, while WM function appears normal (e.g., no significant difference between cases and controls in behavioral data), there is reduced activation in the PFC network – for example in opiate maintenance patients (Bach et al., 2012). Similarly, reduced right parietal cortex activation in cocaine users (Bustamante et al., 2011), reduced ACC and medial PFC activation in adolescent and adult binge drinkers, respectively, (Crego et al., 2010; Bogg et al., 2012) and reduced functional connectivity in frontostriatal networks in cocaine dependent individuals (Ma et al., 2014). Conversely, other studies have reported increased activation when WM performance is not significantly different between cases and controls. For example, increased bilateral supplementary motor area and PFC/dorsal ACC regions occurs in binge drinkers (Vollstädt-Klein et al., 2010; Campanella et al., 2013; Charlet et al., 2014) and increased PFC activation is observed as cannabis use increases (Jager et al., 2010; Cousijn et al., 2014a). Furthermore, increased PFC activation is shown to remain over 3 years in cannabis users (Cousijn et al., 2014b; Goudriaan, 2014). Similarly, increased bilateral insula, medial superior prefrontal cortices, and right precentral gyrus activation has been observed in marijuana users (Schweinsburg et al., 2010). Increased PFC and posterior cingulate cortex activation during WM tasks may be predictive of relapse – at least for nicotine smokers (Loughead et al., 2015), and in chronic but not acute smokers normal WM performance is associated with increased activation of bilateral PFC, temporal, parietal, and insular cortices (Sweet et al., 2010; Sutherland et al., 2011). Interestingly, one study suggests that chronic alcoholics recruit cerebellar function resources to stimulate PFC networks during WM tasks (Chanraud et al., 2013). Finally, structural brain imaging studies when no significant WM deficit is observed, have reported increased basal ganglia volume using MRI after 4 weeks of cognitive treatment for methamphetamine use (Brooks et al., 2016) and increased frontocerebellar volume in those with alcohol use disorder (Chanraud et al., 2010). Taken together, the neuroimaging studies clearly implicate differential neural activation in brain regions that are typically associated with the WM network, including the frontostriatal, parietal, insula cortices, and cerebellar region. However, it is not yet clear how increased or decreased neural activation or volume in these regions differentiates WM function in those with SUD, with studies suggesting compensatory or inefficient mechanisms across networks to enable normal WM performance.

The remaining *n* = 11 (35%) brain imaging studies in our systematic review reported worse WM performance in SUD compared to controls and altered neural processes (Bava et al., 2010; Moeller et al., 2010; Smith et al., 2010; Nulsen et al., 2011; Cservenka et al., 2012; Marvel et al., 2012; Fitzpatrick and Crowe, 2013; Ozsoy et al., 2013; Falcone et al., 2014; Claus and Hendershot, 2015; Liang et al., 2016). In conjunction with worse WM performance, structural studies demonstrated reduced fractional anisotropy (FA) in PFC WM networks and higher FA in visual networks that may be linked to cognitive biases in alcohol and marijuana-using adolescents (Bava et al., 2010). Other structural studies showed alcoholic cerebellar degeneration was related to worse WM performance (Fitzpatrick and Crowe, 2013), and reduced hippocampal volumes in adolescents with alcohol use disorder (Ozsoy et al., 2013), which may alter the solidification of episodic memories within the WM network. In terms of brain function, decreased activation in the superior temporal gyrus and worse WM performance was observed in male alcohol users after an acute dose of alcohol (Claus and Hendershot, 2015). Decreased PFC activation and worse WM was observed in youth with a family history of alcoholism (Cservenka et al., 2012). Similarly, abstinent smokers performed slower on a WM task and had decreased DLPFC and ACC activation, but older age appeared to attenuate the effects (Falcone et al., 2014). Cocaine dependent individuals show reduced frontostriatal activation, and increased thalamus activation in relation to treatment response (Moeller et al., 2010). Ecstasy (MDMA) users during an EEG study also showed reduced electrophysiological indices during a WM task (Nulsen et al., 2011). Another study using SPECT measured another type of brain function – dopamine transportation (DAT), and found reduced DAT levels in the striatum of opioid dependent subjects that related to non-perseverative (e.g., omission) errors during a WM task (Liang et al., 2016). On the other hand, increased activation in inferior/superior cerebellum and amygdala in methadone maintenance opioid dependent participants has been observed (Marvel et al., 2012), and increased activation of the middle/superior frontal gyrus and right superior temporal gyrus in young marijuana users during worse WM performance (Smith et al., 2010). Taken together, worse WM performance appears to imply deficits (reduced/aberrant structure and function) in brain areas linked to the WM network, namely the PFC, hippocampus, temporal gyrus – and also the cerebellum.

Some of the neuroimaging studies in our systematic review included pharmacological challenge paradigms that go one step further in identifying the neural mechanisms of WM in SUD. Eleven pharmacological studies were found, including the use of *tolcapone* (a COMT inhibitor) (Ashare et al., 2013); *methadone substitution therapy* (an opioid derivative) (Bach et al., 2012; Henry et al., 2012; Marvel et al., 2012; Rapeli et al., 2012; Rass et al., 2015); *buprenorphine substitution therapy* (an opioid derivative) (Bach et al., 2012; Rapeli et al., 2012); *modafinil* (a GABA inhibitor that has stimulant properties) (Kalechstein et al., 2010, 2013; Dean et al., 2011; Joos et al., 2013), and *rivastigmine* (an acetylcholinesterase inhibitor) (Mahoney et al., 2014). The study using tolcapone did not provide strong evidence that reducing the function of COMT significantly alters WM function in abstinent nicotine smokers (Ashare et al., 2013), though that is not to say this approach would not work in other SUDs, e.g., stimulant users, in line with altered dopaminergic function. Studies using methadone or buprenorphine opioid substitution therapy showed no significant influence on WM (Bach et al., 2012; Henry et al., 2012; Marvel et al., 2012; Rapeli et al., 2012; Rass et al., 2015). Conversely, there is accumulating evidence that modafinil may significantly improve attention and WM in those with SUD (Kalechstein et al., 2010, 2013; Dean et al., 2011; Joos et al., 2013), and may be a useful pharmacological partner for other interventions aiming to improve WM, such as WM training. Finally, in the only preliminary study using rivastigmine with cocaine dependent participants to date, there was significant evidence that acetylcholinesterase inhibitors may improve WM deficits (Mahoney et al., 2014). Thus, while most pharmacological agents have not proven to be effective at improving WM deficits in SUD, modafinil and rivastigmine may be beneficial in conjunction with other psychological/cognitive interventions, particularly for those with severe cognitive deficits.

Perhaps one of the most prominent findings arising from the systematic review of WM in SUD is that the PFC – implicating dopaminergic function and the dual process model (Goldman-Rakic, 1995, 1998; Bechara, 2005) – is most altered (both increased and decreased activation). Furthermore, PFC activation may moderate the function of other brain regions implicated in our review, namely the cerebellum, insula, basal ganglia (including hippocampus, amygdala, striatum, ACC), thalamus, temporal gyrus, and parietal cortex, in line with contemporary neural models of addiction (Everitt, 2014). From the viewpoint of dopaminergic dysfunction that pertains to cognitive control deficits, an association implicating PFC volume differences has been reported (Genevsky et al., 2010). Furthermore, the link between psychosis and prolonged SUD, particularly in the use of stimulants is established and highlights the role of prefrontal dopaminergic system dysregulation (Bramness and Rognli, 2016). Moreover, effective treatment for psychosis, which is often observed in those with chronic SUD, targets PFC systems involving the DLPFC (Welch et al., 2011; Li et al., 2015; Kani et al., 2016). Similarly, repetitive transcranial magnetic stimulation of the right DLPFC leads to a significant reduction in craving for substances (Enokibara et al., 2016). Related to this, targeted cognitive training for schizophrenia using auditory (as opposed to visuospatial) WM tasks is most effective at reducing symptoms that might coincide with neuroplasticity changes (Biagianti et al., 2016). Thus, combined these findings suggest that PFC dopamine dysfunction in particular, might underlie variations in cognitive control, particularly with regard to auditory/verbal strategies for future goals and modulation of distracting stimuli that may shed light on the role of WM in SUD.

### Genetic Influences on WM in SUD

#### Genetics and Delay Discounting

While there were not enough studies for a systematic review, there is a growing interest in the field of behavioral economics, which considers *delay discounting* an endophenotype of cognitive control deficits in addictive disorders. Delay discounting, broadly synonymous with impulsivity, confers greater value to an immediate – over a delayed reward (for review, see MacKillop, 2013), and one study in our review found that WM training improves delay discounting in stimulant users (Bickel et al., 2011). In terms of neural systems, the PFC is a significant local circuitry to examine with respect to delay discounting and WM, given that GABAergic delay interneurons, influenced by excitatory NMDA receptors, particularly the NRB2 subunits, endow this area of the cortex with “neural psychic properties” (Monaco et al., 2015) to keep in mind goals for future reward. The function of these neurons is a “double-edged sword,” however, given their propensity to neuro-excitotoxicity and psychiatric disorder, particularly schizophrenia (Monaco et al., 2015). In addition, it is suggested that genetic variation related to dopamine neurotransmission is significantly associated with variability in discounting preferences, although the findings are currently in need of replication (MacKillop, 2013). For example, delay discounting has been examined in a sample of nearly 200 participants at high risk for impulsivity in relation to two genetic variants, the DRD2/ANKK13 Taq IA SNP (rs1800497) and the polymorphism in exon 3 of the dopamine D4 receptor gene (DRD4 VNTR) (Eisenberg et al., 2007), a receptor found in PFC. This study reported that A1 allele carriers who also had at least one long version of DRD4 VNTR demonstrated significantly higher levels of impulsive discounting compared to the other genotype combinations (MacKillop, 2013). Furthermore, other studies have examined the COMT val158met SNP (rs4680) in relation to delay discounting. In one study of alcoholics compared to healthy individuals those who were homozygous for the COMT valine variant exhibited significantly more impulsive discounting (Boettiger et al., 2007). Also, in terms of impulsive discounting and the COMT val-val genotype, the val allele is associated with greater enzymatic metabolism of dopamine, which results in fast dopamine degradation that terminates more quickly the actions of this neurotransmitter, and may be associated with impulsivity (Savitz et al., 2006). Taken together, while the genetic data pertaining to delay discounting are mixed, and there are not currently enough studies for systematic review, there is an emerging trend for dopaminergic hypofunction to be associated with increased impulsivity and delay discounting, implicating expression of the dopamine receptor genes (Eisenberg et al., 2007). It is also pertinent to consider that epigenetic effects in terms of DNA methylation in the MB-COMT promoter gene are associated with improved verbal WM function and increased left DLPFC activation in humans (Walton et al., 2014).

#### Epigenetics in SUD

It is useful to consider how to harness epigenetic effects in brain regions that predispose to addiction, given that receptor studies examining potential psychopharmacological agents or psychosocial methods have not curbed the rise in rates of addiction (Cadet et al., 2016). Accumulating evidence implicates dopaminergic epigenetic effects in addiction, and the starting point was that D1 dopamine receptor (predominantly expressed in human PFC) knock-out mice cease to self-administer cocaine (Caine et al., 2007), whereas D3 (predominantly expressed in the striatum) knock-out mice increase their self-administration of the drug (Song et al., 2012). This might suggest that higher levels of synaptic dopamine in the prefrontal cortex curtails addictive behaviors, especially given that lower levels (due to rapid degradation of prefrontal dopamine) is linked to addictive behaviors such as impulsivity (Savitz et al., 2006), although higher cortical dopamine level is also linked to schizophrenia (Schacht, 2016). However, this again hints at an inverted U-shape characterization of dysfunction in the prefrontal cortex according to dopamine levels that are transiently too low or high (Abi-Dargham, 2003; Vijayraghavan et al., 2016). Furthermore, higher basal ganglia synaptic dopamine levels may lead to increased dopamine arriving at the PFC, and less top-down cognitive control of appetitive processes. Specific epigenetic effects that facilitate transmission at dopaminergic synapses might contribute to inverted U-shape variations in function within the dopamine circuitry in those addicted to psychostimulants (e.g., cocaine and amphetamine-like drugs) involving the expression of several histone acetyltransferases (HATs) and histone deacetylases (HDACs) (Cadet et al., 2016). Taken together, if chronic SUD underlying a switch from recreational to controlled to habitual drug taking (e.g., Everitt, 2014), involves epigenetic effects mainly in dopaminergic pathways, then WM training that targets the same circuitry may help to harness and improve dysfunctional epigenetic effects in AN, SUD, and other impulse control disorders.

## Working Memory Training As A Novel Adjunct to Treatment to Improve Cognitive Control

The neuroscience of epigenetics as cognitive enhancers in humans (e.g., to harness and increase cognitive reserve, as related to level of education), particularly when targeting specific brain regions is growing popular in recent years (Mikaelsson and Miller, 2011). Accumulating evidence shows that repetitive training using increasingly difficult (“scaffolding,” Baker, 2010) WM tasks may harness and strengthen inherent neuroplasticity, particularly as a transdiagnostic treatment for SUD (Sofuoglu et al., 2016) in key regions of the human WM network, such as the DLPFC, medial PFC, parietal cortex, insula, and striatum (Li et al., 2015). This may be particularly relevant to those whose early life experiences are detrimental to developmental brain processes (e.g., childhood trauma and fetal alcohol syndrome). However, it must be remembered that the main criticism of the efficacy of WM training is whether near-transfer effects (e.g., improvements on the WM task) translate into far-transfer effects (e.g., improvements in fluid/crystalline intelligence, quality of life and non-related cognitions). Furthermore, when considering the epigenetic effects of WM training in humans that might underlie neuroplasticity mechanisms, there is currently no evidence available, to the authors’ knowledge, and so research into the underlying epigenetic mechanisms of WM training is needed (Keshavan et al., 2014). However, in animal models, WM training, for example, in infant rats, alters learning-induced synaptic plasticity, such that spine formation is suppressed in the ventromedial PFC, whereas spine pruning is suppressed in the lateral orbitofrontal cortex (Bock et al., 2014).

Currently, there are various WM training paradigms on the market, but very few with peer-reviewed evidence to support claims of beneficial effects. Conducting a systematic review of WM training paradigms is outside the aim of the present article, but currently the leader in the field appears to be CogMed^TM^ by Torkel Klingberg (for recent review, see Spencer-Smith and Klingberg, 2015), with *n* = 52 publications relating to CogMed to date. CogMed^TM^ is an online battery of WM training tasks supported by qualified professionals all over the world, mainly specializing in improving both near and far-transfer with relation to attention and impulsivity in children, adolescents and adults with ADHD and related attention difficulties. CogMed^TM^ has also proven beneficial to other populations, including healthy school children who improve their academic performance with WM training (Bergman Nutley and Söderqvist, 2017); as well as children with traumatic brain injury (Phillips et al., 2016).

In line with the hypothesis that repetitive use of WM processes evokes neuroplasticity in the corticostriatal circuitry, we have recently shown that 4 weeks of increasingly difficult WM training (using a smartphone app developed by Dr. Brooks: ‘Curb Your Addiction: C-Ya’) in participants with SUD evokes widespread increase in bilateral basal ganglia volume (incorporating the amygdala and hippocampus) (Brooks et al., 2016). Given that we reported an average learning rate of 35% on the most difficult level of the *N*-back training during the study (3-back) coinciding with improvements in self-reported impulsivity scores, we suggest that increased basal ganglia volume may reflect dopaminergic epigenetic effects, although epigenetic effects were not measured in the study. This notion is supported by a recent animal study demonstrating that WM training triggers delayed chromatin remodeling in the mouse cortico-striato-thalamic circuit (Cassanelli et al., 2015). Specifically, increased PFC and dorsomedial striatum activation as measured by c-fos, a neuronal marker of activation was reported by Cassanelli et al. (2015). Additionally, they observed epigenetic effects of WM training in terms of induced late changes in both H3 methylation and acetylation in the dorsomedial striatum and the dorsomedial thalamus, but not in the PFC (Cassanelli et al., 2015). However, epigenetic effects in the basal ganglia may reflect downstream modulation by PFC, although the inverted U-shape nature of rapid regulation of receptors in the PFC may suggest that these effects are more difficult to observe.

With this in mind, humans engaging in WM training demonstrate increased prefrontal and striatal dopaminergic activation (D’Ardenne et al., 2012; Tanida et al., 2012), and WM training has been shown to improve impulsivity in patients with SUD (Bickel et al., 2011). In this vein, a recent meta-analysis of fMRI studies of WM training in people with SUD demonstrated that, in line with reduced delay discounting there is increased bilateral DLPFC activation (Wesley and Bickel, 2014). To date, however, there have been no studies examining whether WM training reduces compulsive and repetitive ruminations, and related changes in neural processes in people with AN. In fact, compared to SUD, as our systematic review confirms, there is a paucity of research examining WM mechanisms and their relation to excessive cognitive control in AN. Thus, against the background of burgeoning neurobiological evidence into WM processes and the potential beneficial effects of WM training implicating the corticostriatal network (for review, see Spencer-Smith and Klingberg, 2015), it appears that it is a worthwhile research field to pursue in relation to improving cognitive control of impulsivity and prognosis for psychiatric disorder, but there are still many questions left unanswered.

## Implications and Future Questions to Be Answered

This review article has used the impulse control spectrum model (Brooks et al., 2012b; Brooks, 2016) to compare the role of WM in cognitive control in AN and SUD. To consider the neurobiology of WM (Baddeley and Hitch, 1974), we included reference to contemporary theories of WM in the healthy human brain that resonate with dual process theory of impulse control (Bechara, 2005; Sofuoglu et al., 2016), namely Global Workspace Theory (Baars et al., 2013), and Bayesian Probabilistic Inference, or the Bayesian Brain (Nilsson, 1986; Friston et al., 2015). As such, before a systematic review of WM studies in AN and SUD, we attempted to conceptualize the role of WM in the transient experience of cognitive control. Within this conceptualization, we have described how backstage saliency processes supported by the dopaminergic basal ganglia network are activated non-consciously and perhaps half a second prior to the decision to exercise cognitive control (e.g., “free-won’t” or conscious veto, Libet, 1985, such as appetite suppression in AN). We have further described a minimum threshold of balanced activation between interneurons in the GABAergic and glutamatergic PFC circuits. The activation, which is likely transient and related to meta-assemblies of functionally decaying neuronal clusters that adhere to an inverted U-shape pattern, whereby visual representations of future goals/predictions are held in mind in the absence of external stimulation (Yantis, 2000). Then, we described some research into how increasing the cognitive load of WM and PFC activation may strengthen the neuronal architecture of these circuits and reduce the competitive interference of bottom-up distracting affective stimuli.

Against the background of healthy human WM brain function, we conducted two systematic reviews of WM processes in AN and SUD. By doing so, we related reviewed studies to data in adolescents with an acute – and adults with a chronic diagnosis of AN, whereby obsessive-compulsive rumination tendencies reflect epistemic foraging and are likely supported by excessive and rigid WM performance (particularly in the audio/verbal domain). Furthermore, this pattern of WM function likely alters neuroplasticity in DLPFC and the related corticolimbic network (including the parietal, temporal, and insular cortices). We utilize the dual process addiction model (Bechara, 2005) to describe AN (O’Hara et al., 2015) in terms of how corticostriatal reward circuits may be hijacked by rumination restraint cognitions, switching from deliberate to habitual dieting behavior. We then explored epigenetic effects implicating dopaminergic pathways in AN, involving the synaptogenic BDNF and SOX2-OT gene that may play a part in neurogenesis and transient dopaminergic transmission within the WM network. We described that genetic susceptibility in AN confers risk for schizophrenia and bipolar disorder, but protects against developing SUD. We then systematically reviewed studies of SUD at the opposing end of the impulse control spectrum model (Brooks et al., 2012b; Brooks, 2016), in terms of verbal WM deficits (phonological loop) and a switch from controlled prefrontal cortex and ventral striatum activation to habitual dorsal striatum activation. Then, we described how genetic susceptibility and epigenetic effects within dopaminergic corticolimbic pathways are associated with SUD. Thereafter, we concluded by briefly describing some empirical studies regarding WM training and the influence on neural processes. Finally, we now briefly address some remaining questions and suggest novel experimental methods for future research that may provide some answers.

### Questions Unanswered and Novel Experimental Methods for Testing the Neurobiological Model of WM and Cognitive Control

**FIGURE 5:**
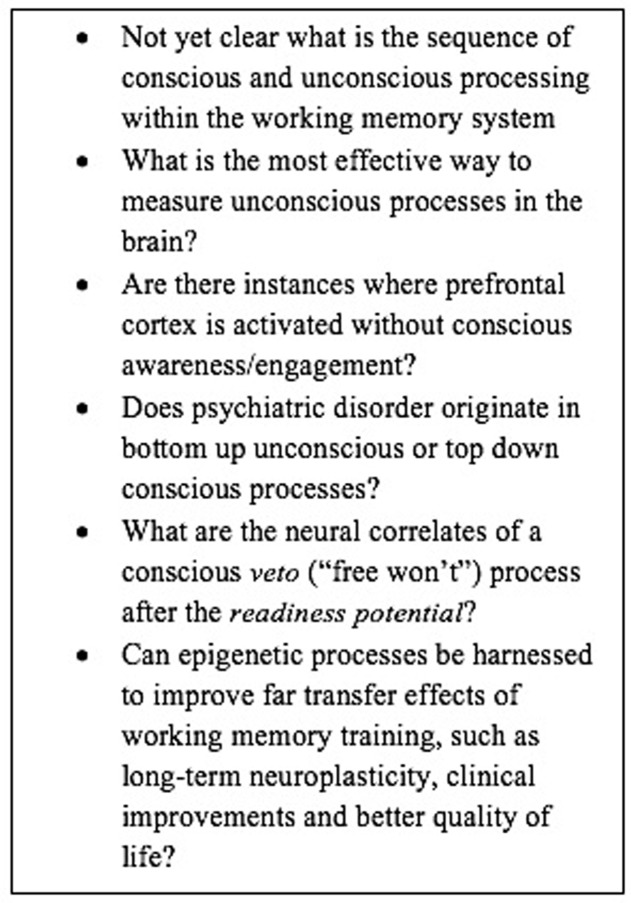
Summary of the research questions still to be answered in the field of neural correlates of WM and cognitive control.

Various questions remain unanswered as to the neurobiological underpinnings of cognitive control and the link to WM in the healthy brain, and in AN and SUD, for a summary, see **Figure 5**.

Firstly, it is not yet clear whether unconscious processing is the first step in the sequence of cognitive control within the WM system, whether deliberative metacognitive processing is the key, or if it is a circular relationship between cognitive and affective processes, which are modified over time (Shea et al., 2014). As such, it is not clear how the WM system interacts with the mesolimbic circuitry to forge cognitive control of impulses, although it is likely related to storage, encoding and retrieval of future goals and prior beliefs that are stored in the episodic buffer via the hippocampus. Secondly, it is not yet clear what is the most effective way to measure unconscious processes in the brain, and so far, backward masking (**Figure 4**), event-related potentials with EEG (**Figure 3**) and subliminal tasks with fMRI (Brooks et al., 2012c) have been commonly used, but there are other novel experimental methods to consider as described below. Thirdly, given that there is some evidence that the ACC, part of the corticolimbic network, is activated to non-consciously processed stimuli (e.g., Brooks et al., 2012c), it is not yet clear if there are instances where the PFC is activated without conscious awareness/engagement. For example, compulsive repetitive restraint cognitions in those with AN may become unconsciously activated in the PFC, but these may be driven by alterations in bottom-up saliency network processes. Fourthly, it is not yet known whether psychiatric disorder, pertaining to deficits in WM and cognitive control, originates in excessive bottom-up saliency networks, or aberrant top-down conscious metacognitions, or whether both are important. Fifthly, while there is some evidence implicating the dorsal fronto-medial cortex in terms of a conscious veto (“free won’t”) of anticipated behavior (e.g., eating or substance use) (Kühn et al., 2009), which is linked to reduced EEG readiness potentials during voluntary omissions but not rule-based omissions (Misirlisoy and Haggard, 2014), the field remains relatively unexplored. Finally, it is not yet clear whether epigenetic processes can be harnessed to improve cognitive control and far transfer effects of WM training, such as long-term neuroplasticity, clinical improvements and better quality of life. Related to this, it is not known whether WM training can alter regulation of GABAergic and glutamatergic receptors that contribute to cognitive control and reduced delay discounting via PFC activation that may be associated with AN.

Attempts to answer these unyielding questions could consider novel non-invasive experimental approaches, such as, but not limited to, repetitive Transcranial Magnetic Stimulation (rTMS), real-time fMRI (rt-fMRI) and imaging epigenetics. Firstly, a recent meta-analysis demonstrates that rTMS, which is suggested to increase activation in the right DLPFC and has the potential to alter receptor regulation and neurotransmitter function significantly improves craving in those with SUD (Enokibara et al., 2016). Similarly, rTMS of the DLPFC has mixed findings in those with binge eating disorder (Van den Eynde et al., 2010; Gay et al., 2016) but is nevertheless linked to enhanced frontostriatal connectivity and reduced craving (Dunlop et al., 2015). However, rTMS of the DLPFC improves AN symptoms linked to more prudent decision-making (McClelland et al., 2016) and improves WM performance in general (Brunoni and Vanderhasselt, 2014). With these rTMS studies in mind, it is useful to consider that lateralization of function in the DLPFC has been reported, in that the left DLPFC is linked to future control, whereas the right DLPFC is linked to immediate control (Vanderhasselt et al., 2009). Against this background, future studies could examine whether a combination of increasingly difficult WM training and rTMS over the left or right DLPFC could foster long-term changes in the corticolimbic dopaminergic circuitry that translate into clinical and quality of life improvements.

Secondly, rt-fMRI explores biofeedback mechanisms such that participants learn, via conditioning, the association between physiological experience of cognitive control and visual representation of brain activation (Val-Laillet et al., 2015). However, while emerging evidence supports neurofeedback-guided upregulation of hypoactive networks, it appears more difficult to downregulate hyperactive networks (Ihssen et al., 2016). Nevertheless, some recent evidence suggests that motivational neurofeedback can reduce the incentive salience of appetitive food cues, and that this is linked to alterations in the corticolimbic circuitry (Ihssen et al., 2016). Furthermore, Ihssen et al. (2016) conclude that decreased neural responses to salient stimuli appears not to be regulated by top-down processes, but rather arises from subcortical regions related to implicit operant reinforcement of brain activity. Similarly, for SUD (alcohol), rt-fMRI has been shown to be effective in reducing functional connectivity between ACC, insula (limbic saliency network), inferior temporal gyrus (part of the “what” visual pathway, and may indicate reduced cue-induced saliency) and medial frontal gyrus (associated with self-related processing) (Karch et al., 2015). Thus, against this background, rt-fMRI might positively reinforce a participant involved in WM training, by demonstrating how improved WM performance, and thus cognitive control, is linked to neural activation.

Finally, examining epigenetic variations that are related to gene methylation in peripherally assessed DNA (e.g., blood and saliva) and correlating with behavioral and clinical measures associated with brain function is a new field that aims to locate biological mechanisms of risk for psychiatric disorder (Nikolova and Hariri, 2015). It is suggested that the first steps in the field of imaging epigenetics should examining well-established neural circuitry, such as dopaminergic genes and the reward/motivation pathways (Schultz, 2002) and by combining this approach with multi-modal brain imaging techniques (e.g., positron emission tomography, EEG, fMRI) (Nikolova and Hariri, 2015). Furthermore, it is suggested that epigenetic imaging should be guided by the most significant GWAS data, and in terms of AN and SUD the dopaminergic system (BDNF, DAT, and DAD2) and also the SOX2-OT gene associated with neurogenesis are implicated. Specifically, in terms of AN, while most recent GWAS studies do not support the significant data found in single studies, the opioid, leptin, ghrelin, orexin, and serotonin receptor genes may be promising areas of further exploration for epigenetic imaging studies (Gorwood et al., 2016). Specifically, in terms of SUD, there is an urgent need for biomarkers that are associated with chronic addiction to be classified, but presently the dopaminergic reward system appears to be key (Volkow et al., 2015).

## Conclusion

The major suggestion, or ‘red line’ throughout this article was that WM capacity, which supports the verbal repetition of cognitive strategies that aid in the experience of cognitive control and epistemic foraging for beliefs about the (uncertain) future, may not be limited to ‘seven plus or minus two’ (Miller, 1956) but can be widened, deepened, strengthened or made more flexible by repetitive use of WM. If this is the case, then WM training may be a useful adjunct to improve WM, promote neuroplasticity changes and enhance treatment effects in those with impulse control disorders. WM has a long history of being associated with cognitive control (e.g., Goldman-Rakic, 1995, 1998; Bechara, 2005) and supports healthy epistemic foraging of information from internal or external cues that help guide decisions and behavior. The link between WM and epistemic foraging reflects the Bayesian view of brain processing, where bits of information are transiently kept in mind to update our prior beliefs about the world. If more bits of information can be held in mind (e.g., with greater WM capacity) then it might be that better prediction updating occurs based on prior beliefs. Similarly, the Bayesian brain view posits that too much uncertainty (e.g., a suboptimal cognitive model, perhaps related to limited WM capacity) can lead to greater allostatic load, stress and anxiety, which are common comorbidities in AN and SUD.

And so, it holds that a larger (or more flexible) WM capacity could support more bits of information that are transiently evaluated in the mind for improved updating of prior beliefs. This might prevent the bias of ‘jumping to conclusions’ as is sometimes observed in those with SUD, or rigid, local versus global cognitive processing as often occurs in those with AN (Kothari et al., 2013). However, when WM capacity is smaller, or dysfunctional, this might translate into heightened allostatic load and psychiatric disorders such as AN or SUD. To test this hypothesis, after detailing healthy neuropsychological, neural and genetic processes underlying WM, we have highlighted varying degrees of cognitive control and differences in WM by systematically reviewing AN and SUD. We have compared studies of AN and SUD because they have previously been associated with extremes of cognitive control on an impulse control spectrum model of eating disorders, whereby normal control is in the middle (Brooks et al., 2012b; Brooks, 2016) (**Figure 1**) and SUD has similarities to binging (Volkow and Baler, 2015). Using an *a priori* model upon which to test a hypothesis and update a theory is a robust and replicable scientific method.

Using the impulse control spectrum model, we have examined the neuropsychology, neural and genetic processes underlying WM and its role in cognitive control in AN versus SUD. From a neuropsychological perspective, there are mixed findings with regard to WM in AN, but the data are suggestive of over-compensatory mechanisms to achieve a normal WM ability. In terms of the neuropsychology of WM in those with SUD, more studies have been conducted and suggest that there is a weakening of performance (e.g., reduced capacity), that is exacerbated by an inability to avoid distractions and impulsively choose immediate over delayed rewards. In terms of the neural mechanisms of WM in AN, excessive activation of the ECN (e.g., DLPFC, ACC, interacting with limbic regions) appears to be key, and may be a trait, as opposed to a state of the disorder. Conversely, those with SUD appear to have excessive activation of the mesolimbic reward pathway – also known as the saliency network – and dysfunctional activation of PFC networks. Finally, in terms of (epi)genetic findings, for those with AN there is inconclusive evidence at present, whereas for those with SUD genetic linkage with the dopaminergic brain systems (e.g., receptors transporters, and enzymes) might be key. And while no epigenetic studies have yet examined the dynamic influences on genetic expression in AN or SUD, animal studies hint that delay interneurons and a discrete balance between activation of glutamatergic and GABAergic PFC circuits (in the absence of external stimuli) may foster different WM profiles that may link to variations in cognitive control. As such, chronic hyper- or hypo-activation of ECNs, in conjunction with variations in activation in the mesolimbic salience pathways, may foster changes in receptor regulation that contribute to changes in cognitive control of impulsivity at the neural level.

**FIGURE 6:**
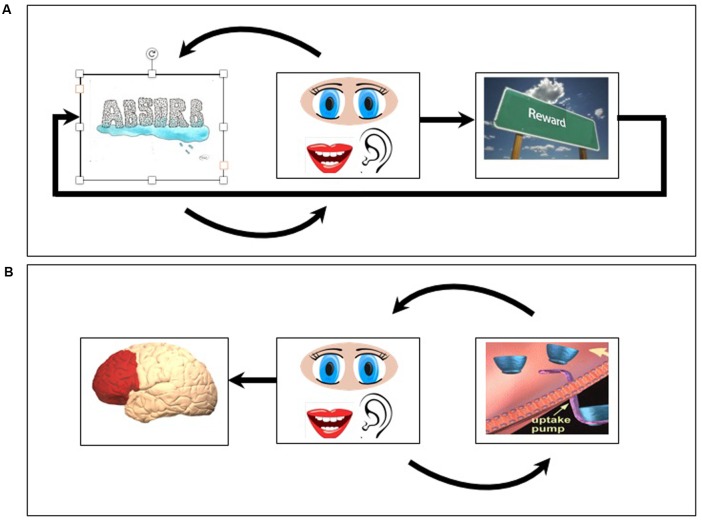
Schematic diagram of the dopamine absorber and dopamine pump analogy. Image of dopamine uptake pump by permission from http://www.nida.nih.gov/pubs/teaching/teaching2/Teaching2.html. Image of eye by permission from http://www.clipartpanda.com/categories/blue-eyes-clipart. Image of ear by permission from http://www.clipartkid.com/ear-cliparts/. Image of mouth by permission from http://www.clipartkid.com/mouth-cliparts/.Image of “1” reward badge by permission from http://www.clker.com/cliparts/B/Z/b/k/v/7/award-symbol-md.png. Image of red prefrontal cortex by permission from https://commons.wikimedia.org/wiki/Category:Prefrontal_cortex#/media/File:Prefrontal_cortex_(left)_-_lateral_view.png. **(A)** Dopamine absorber analogy (anorexia nervosa): excessive, deliberative activation of the prefrontal cortex (sponge), by way of repetitive cognitive ruminations, absorbs increased endogenous dopamine release via the basal ganglia (reward symbol) via epigenetic upregulation of receptor systems. Repetitive phonological and visuospatial rehearsal (eyes, ears, mouth), held in mind by prefrontal cortex delay interneurons strengthens the saliency (and therefore the ability to stimulate basal ganglia dopamine release) and relevance of imagined images. To prevent habituation over time, imagined images become more detailed and complex to stimulate required levels of dopamine for prefrontal cortex absorption. **(B)** Dopamine pump analogy (substance use disorder): excessive activation of basal ganglia dopaminergic system (uptake pump) by way of consumption of substances increases the release and re-uptake of dopamine. This in turn biases repetitive phonological and visuospatial rehearsal (eyes, ears, mouth) in favor of the stimulus that is associated with increased dopamine release (e.g., food and drugs). Excessive dopamine arriving at the prefrontal cortex weakens top-down control and encourages exogenous dopamine release stimulation. In both analogies, there is a propensity for increased dopamine in the prefrontal cortex, which leads to epigenetic effects, inflexible cognitive style and chronic disorder, as well as risk for the development of psychosis.

In order to bring together, in a simple visual, the potential mechanisms of suboptimal WM in AN and SUD proposed here, one could use the analogies *dopamine absorber* versus *dopamine pump*, respectively (**Figure 6**). In both cases, there is a lack of cognitive flexibility as excessive dopamine release biases neural responses. In the dopamine absorber system (AN), neural activation associated with excessive WM function and eating disorder ruminations hijacks corticostriatal dopaminergic pathways, such that PFC epigenetic upregulation of dopaminergic, glutamatergic, GABAergic receptors occurs in response to higher levels of basal ganglia dopamine release. Repetitive and increasingly detailed local – as opposed to global rumination on auditory eating disorder mantras over an extended period of time (e.g., “I will only eat salad at noon”), as per the WM model, solidifies images in hippocampal episodic memory networks, such that the saliency of these images, and their ability to activate dopamine systems, becomes stronger and non-conscious, particularly in conditions of uncertainty. Epigenetic upregulation of GABAergic and glutamatergic antagonistic networks within the PFC, in the absorber analogy, foster greater activation of delay interneurons that supports more detailed epistemic foraging. This could be synonymous with strengthened cognitive control in response to increased incentive saliency for imagined stimuli and predictions about future goals (Yantis, 2000).

Conversely, in the dopamine pump system (SUD), ingestion of rewarding substances, akin to binge eating (Volkow and Baler, 2015), bypasses prefrontal cortex modulation, such that downregulation of receptors in the basal ganglia dopaminergic network confers risk for craving and reduced (or biased) epistemic foraging. Disruption, as a result of greater bottom-up dopamine availability following substance use, weakens the ECN that underlies WM. In both the dopamine absorber and dopamine pump scenarios there is a heightened risk for schizophrenia, but more so in SUD, given that there is an increase in PFC dopamine flooding the WM system and promoting a model-free, ‘jumping to conclusions’ scenario. This would hinder optimal inference generating and epistemic foraging of reality to update prior beliefs. Furthermore, the experience of early life adversity, which is perhaps more common in those with SUD than AN, may have detrimental effects on the WM system that supports the exercising of prior beliefs about the self, world and others, which particularly involves the cortical to hippocampal circuitry.

In line with the findings of this review, increasingly difficult repetitive WM training may harness inherent epigenetic effects that underlie neuroplasticity within the corticolimbic dopaminergic system, such that improvements may occur in the use of cognitive control over impulsivity (which is related, more broadly, to fluid intelligence), and related far-transfer effects (e.g., improvements to attention and hyperactivity). WM training may also aid those with AN by widening WM capacity that may support greater flexibility, less attention to detail and global versus local cognitive processing. Refined measurement of the neural systems underlying WM and its link to cognitive control might better test the assumptions of the impulse control spectrum model, more broadly to fit not only eating disorders but also those with addiction, to strengthen interventions for disorders such as AN and SUD.

## Author Contributions

SB conceived and wrote the article; HS checked and suggested improvements to the manuscript; SF and SY helped to systematically reviews studies and checked the manuscript.

## Conflict of Interest Statement

The authors declare that the research was conducted in the absence of any commercial or financial relationships that could be construed as a potential conflict of interest.
