# Supplementary material for: The Role of Working Memory for Cognitive Control in Anorexia Nervosa versus Substance Use Disorder
Source: Front Psychol. 2017 Sep 22;8:1651. doi: 10.3389/fpsyg.2017.01651 (PMC5615794; doi:10.3389/fpsyg.2017.01651)
Supplement: Supplementary file 1 [file Table_1.PDF]

Table 1. Alphabetical list of studies examining WM and cognitive control in anorexia nervosa

Author (year): worse WM performance in red font<br>Title of study: brain imaging studies in green font	Participant details	Experiment details	Main outcomes	Implications
Anorexia Nervosa studies=15; number of total AN participants: n=582; number of total HC: n=365				
Bentz et al., (2017)<br><br>*Neurocognitive functions and social functioning in young females with recent-onset anorexia nervosa and recovered individuals*	N=43 young females with first-episode AN<br><br>N=28 individuals recovered adolescent-onset AN<br><br>N=41 control individuals<br><br>(mean age:14-22 yrs)	All groups were measured across 7 neurocognitive functi ons: set-shifting, local processing, processing speed, working memory, sustained attention, verbal memory, and verbal abstraction.	Participants did not differ across groups. Verbal memory corresponded to better social functioning.	Young women either with or recovering from AN did not appear to have substantial cognitive deficits. Verbal memory appeared to be related to social cognition.
Biezonski et al., (2016)<br><br>*Evidence for Thalamocortical Circuit Abnormalities and Associated Cognitive Dysfunctions in Underweight Individuals with Anorexia Nervosa*	N=28Female underweight adults with AN (11 binge AN 17 Restricting AN) mean age: 19yrs<br><br>N=22 Female HC mean age 21 yrs	Stroop task (for cognitive control of distracting information)<br><br>Letter-number sequencing task (for working memory)<br><br>MRI for thalamic surface formations	AN patients had thalamic deformations and abnormal functional connectivity between the thalamus and the dorsolateral and anterior prefrontal cortices relative to HC.<br><br>Differences in thalamo-frontal connectivity were related to deficits on tasks probing cognitive control and working memory	Underweight individuals with AN have deformations in the thalamofrontal networks that may be related to cognitive deficits in cognitive control and working memory

		Resting state connectivity between fronto-thalamic circuits		
Brooks et al., 2014b)<br><br>*Obsessive-compulsivity and WM are associated with differential prefrontal cortex and insula activation in adolescents with a recent diagnosis of an eating disorder*	All females mean age 15yrs.<br><br>N=20 HC<br><br>N=15 Adolescent EDNOS	Computerised N-back task during fMRI. Self-report measures: Eating Disorder Exam (EDEQ), Barratt Impulsivity Scale (BIS), Hospital Anxiey and Depression Scale (HADS), Obsessive-Compulsive Inventory (OCIR)	OCIR total scores predicted brain activation during the N- back in the left medial prefrontal cortex, anterior cingulated and dorsolateral prefrontal cortex, as well as the cerebellum and insula. None of the other measures correlated with brain activation during the task.	Obsessiveness and compulsiveness are strong predictors of frontal activation in areas linked to top-down control and rumination in adolescent females with an early ED diagnosis.
Brooks et al., (2012a)<br><br>*Subliminal food images compromise superior WM performance in women with restricting anorexia nervosa*	N=20 (mean age 22 yrs.) HC females<br><br>N=13(mean age 25 yrs.) RAN females	Self-report measures: EDI;<br>HADS, self-rated performance<br>Cognitive tasks: N-Back WM task (1-back, 2-back) and Go/No-Go Task with subliminal (masked) presentation of IAPS food, aversive and neutral images	During the N-back task (associated with DLPFC network activation) subliminal food (and not aversive or neutral) images interfered with task performance. Levels of anxiety correlated with this interference effect. However, this interference effect was not observed during the Go/No-Go task (associated with ACC network activation).	Arousal networks (e.g. in basal ganglia) interact with DLPFC and may underlie top-down control in females with restricting anorexia, particularly when experience of anxiety is high.
Castro-Fornieles et al., (2010)	N=14 children and adolescents with AN:	Eating Attitudes Test (EAT-40)	Before treatment, the AN group had significantly higher activation than HC	Hyperactivation in the parietal and temporal lobe during a working memory

A cross-sectional and follow-up functional MRI study with a working memory task in adolescent anorexia nervosa.	(N=12 girls and 2 boys, aged 11–18)<br><br>N=14 HC children and adolescents (7 boys, 7 girls) of the same age	Childrens Depression Inventory (CDI)<br><br>N-back task (1 back versus 0-back) in fMRI	in temporal and parietal areas. During the N-back task the temporal superior gyrus was significantly active. After 7 months of treatment there was a reduction in this brain activation in those with AN.	task, is associated with greater effort to perform within the normal range – and these activations correlate with clinical measures.
Giombini et al., (2016)<br>*Evaluation of individual cognitive remediation therapy (CRT) for the treatment of young people with anorexia nervosa.*	N=92 female participants diagnosed with AN aged between 11 and 17 (mean 15 yrs), receiving Cognitive Remediation Therapy (CRT) at a specialist inpatient unit.	Rey-Osterrieth Complex Figure test (ROCFT), Behaviour Rating Inventory of Executive Function-Self-Report (BRIEF-SR), D-KEFS Colour-Word Interference Test (CWT).	Significant improvement after CRT in Central Coherence Index, Immediate Recall, Cognitive Shift, Behavioural shift, Emotional Control, Working Memory, Plan/Organize, Monitor	CRT helps to improve cognitive deficits in young people with AN
Hatch et al. (2010)<br>*In first presentation adolescent anorexia nervosa, do cognitive markers of underweight status change with weight gain following a refeeding intervention?*	Female adolescents<br>N=37 AN<br>N=45 HC	IntegNeuro-computerized Battery using the N-back continuous performance test of sustained attention.	During underweight status, AN patients had superior WM capacity in comparison to HC.	ED symptoms or comorbidities did not correlate with WM performance.

Israel et al., (2015)<br><br>*N-back task performance and corresponding brain-activation patterns in women with restrictive and bulimic eating-disorder variants: preliminary findings*	All females<br>N=19 ED-R<br>N=27 ED-BP	N-back task with variable cognitive load (arithmetic) and stress (positive and negative feedback) during fMRI acquisition.	ED-R performed consistently better than the ED-BP group on all N-back versions.<br>Further, the ED-R group had increased right DLPFC and premotor cortex activation during the 2-back vs. 0-back task in comparison to ED-BP. ED-BP had weaker WM activation than ED-R.	WM is poorer in eating-disordered individuals with binge-eating/purging behaviors than in those who solely restrict food intake, and that observed performance differences coincide with interpretable group-based activation differences in a frontal region
Lao-Kaim et al., (2014)<br><br>*Functional MRI investigation of verbal Working Memory (vWM) in adults with anorexia nervosa*	Female adults with ED:<br>N=31 AN<br>N=31 HC	N-back task (0, 1, 2 and 3 back). The authors specifically examine verbal WM, incorporating the phonological loop, the phonological store, sub-vocal rehearsal and the central executive, during fMRI acquisition.	No significant difference in WM task performance. All groups showed increased activation in the bilateral IPL, bilateral middle and superior frontal gyri extending into the DLPFC, left precuneus and right insula. The AN group additionally showed positive trends in the left middle temporal gyrus, right precuneus and left IFG.	Although there was a trend towards lower accuracy as duration of illness increased, this was not correlated with activity in regions associated with vWM. These findings suggest that vWM in AN is as efficient and performed using the same cognitive strategy as HC.
Nikendei et al. (2011)<br><br>*Memory performance in acute and weight-restored anorexia nervosa patients*	Female adults:<br>N=34 R-AN<br>N=19 BP-AN<br>N=16 WS-AN<br>N=30 HC	Wechsler Memory Scale Revised (WMS-R) – Digit span backwards.	Currently ill and weight-restored<br>AN patients did not differ significantly from healthy controls with respect to WM. However, there was impaired immediate and	ED symptoms or comorbidities did not correlate with WM performance.

			delayed verbal recall performance in acute AN patients that was found irrespective of AN subtype, and that persisted in weight-restored AN patients	
Phillipou et al., (2015) *Comprehensive neurocognitive assessment of patients with anorexia nervosa.*	N=26 adults with AN (mean: 23 yrs)<br><br>N=27 adult HC (mean: 22 yrs)<br><br>All matched for IQ and age	A neuropsychological battery including speed of processing [Brief Assessment Of Cognition In Schizophrenia: Symbol Coding, Category Fluency: Animal Naming (Fluency) and Trail Making Test: Part A], attention/vigilance [Continuous Performance Test - Identical Pairs (CPT-IP)], working memory [Wechsler Memory Scale (WMS®-III): Spatial Span, and Letter-Number Span (LNS)], verbal learning [Hopkins Verbal Learning Test - Revised], visual learning [Brief Visuospatial Memory Test - Revised],	Significantly slower reaction times for false alarm responses on the continuous performance task in AN (but no significant differences in WM)	The authors suggest that those with AN have a poorer capacity to manipulate and process visuospatial material.

		reasoning and problem solving [Neuropsychological Assessment Battery: Mazes], and social cognition [Mayer-Salovey-Caruso Emotional Intelligence Test: Managing Emotions].		
Pruis et al., (2012)<br><br>*Recovery from anorexia nervosa includes neural compensation for negative body image*.	All female adults<br>N=15 Recovered AN (Mean age: 39 yrs)<br>N=16 HC (mean age: 32yrs)	Brain activation associated with the disruption of WM by images of bodies	Negatively rated images were more disruptive to WM than neutral or positively rated images in both groups; however, amygdala and fusiform activation were greater in women who had recovered from AN than in controls when viewing images of bodies during the working memory task. There were no group differences in lateral prefrontal activity. However, there was more suppression of medial prefrontal cortex activity in women who had recovered from AN in comparison to controls when negatively rated	Recovery from AN is not achieved by dampening an amygdala mediated emotional response to bodies, but instead by developing compensatory neural mechanisms that prevent emotional responses from disturbing cognition.

			images were presented during the working memory task.	
Ritschel et al., (2017)<br><br>Neural correlates of altered feedback learning in women recovered from anorexia nervosa.	All females<br>N=31 recAN<br>N=31 healthy controls (HC)<br>Mean age: 22yrs	Probabilistic Reversal Learning (PRL) Task during fMRI	Increased fronto-parietal network activity in recAN during the PRL task	The neural correlates of cognitive control are still altered in recovered AN, suggestive of a trait – as opposed to a state – effect. Aberrant dorsal anterior cingulate cortex response to negative feedback may relate to the underweight state in AN. However, impaired behavioural adaptation and elevated activation of cognitive control regions in recAN may relate to altered neural efficiency
Solstrand Dahlberg et al., (2017)<br><br>*Adolescents newly diagnosed with eating disorders have structural differences in brain regions linked with eating disorder symptoms.*	N=15 adolescent females recently diagnosed with ED,<br><br>N=28 HC females<br><br>Mean age: 15yrs	Structural MRI<br><br>EDE-Q<br>N-back task	Smaller left superior temporal gyrus in adolescents with ED compared to HC, which correlated with ED cognitions (concerns about eating, weight, and shape).<br><br>Slower working memory reaction time correlated with larger insula volumes in ED participants, but not HC.	Young, newly diagnosed females with ED had volumetric variations in temporal, insula and cerebellar volumes linked to ED cognitions, obsessions, and working memory.

			In ED, higher restraint and obsession scores were correlated with smaller temporal gyrus volumes, and larger cerebellar and striatal volumes.	
Weider et al., (2015)<br><br>*Neuropsychological function in patients with anorexia nervosa or bulimia nervosa.*	Female adults with ED:<br>N=40 AN<br>N=39 BN<br>N=40 HC	WM Index (WAIS-III Manual) Paced Auditory Serial Addition Test 3, 2 Letter Number Sequencing, Digit Span, WMS-R (Spatial Span)	The AN group had lower WM scores than both BN and HC. Lowest lifetime BMI and depressive symptoms explained the worse WM performance in the BN group but not the AN group.	Longitudinal studies are needed to identify the importance of weight restoration and treatment of depressive symptoms in the prevention of a possible cognitive decline.

AN=Anorexia nervosa: RAN=Restricting AN; BN=Bulimia Nervosa; ED=eating disorder; EDNOS= eating disorder not otherwise specified; HC=Healthy Control; MRI=Magnetic Resonance Imaging; WM=working memory; WAIS=Wechsler Adult Intelligence Scale; EDE-Q=Eating Disorders Examination Questionnaire; OCIR=Obsessive-compulsive inventory revised; DLPFC=dorsolateral prefrontal cortex; IFG=inferior frontal gyrus;
