# Supplementary material for: The Role of Working Memory for Cognitive Control in Anorexia Nervosa versus Substance Use Disorder
Source: Front Psychol. 2017 Sep 22;8:1651. doi: 10.3389/fpsyg.2017.01651 (PMC5615794; doi:10.3389/fpsyg.2017.01651)
Supplement: Supplementary file 2 [file Table_2.docx]

## Table 2. Alphabetical list of studies examining WM and cognitive control in SUD

| Author (year): worse WM performance in red font  Title of study: brain imaging studies in green font | Participant details | Experiment details | Main outcomes | Implications |
| --- | --- | --- | --- | --- |
| *Substance use disorder studies: n=93; number of total SUD participant: n=9106; number of total HC: n=3028* | | | | |
| Albein-Urios et al., (2012)  *Comparison of impulsivity and working memory in cocaine addiction and pathological gambling: Implications for cocaine-induced neurotoxicity.* | N=29 Cocaine dependent individuals (CDI) mean age 33yrs  N=20 HC; mean age 36yrs | A case–controlled observational design was used to measure the impulsivity (UPPS-P trait impulsivity, delay discounting), executive functioning (inhibition; Stroop) and WM performance (N-back) of the different groups. | Results found that CDI had higher scores on the UPPS-P Negative Urgency as well as poorer WM performance (2-back scores) in comparison to PG. PG overall had a higher delay-discounting measure. Both the PG and CDI groups had higher Positive Urgency and lower Stroop scores compared to controls. There was also a negative relationship between cocaine usage and WM performance and response inhibition. | -The higher impulsivity scores of CDI and PG may suggest a vulnerability to addiction.  - Due to cocaine’s neurotoxicity effects, there is cocaine-specific deficits related to WM and inhibition. |
| Arias et al., (2016)  *Neurocognitive, psychiatric, and substance use characteristics in opioid dependent adults.* | N = 38 opioid-dependent adults  (aged: 8–68 years) | A cross-sectional study looking at the neurocognitive functioning, psychiatric manifestations and substance use pattern of adults seeking buprenorphine treatment | It was found that 39% (n = 15) of these patients had global cognitive functioning impairment and over one third had other more specific learning deficits (specifically learning and memory). Lifetime alcohol dependence was correlated with deficits in global cognitive functioning, motor functioning and executive functioning. Lifetime cocaine dependence was correlated with motor and executive functioning impairments. | The findings suggest that opioid-dependent adults may require support in decision making owing to their deficits in executive functioning (crucial for decision making) and learning and memory deficits (negative impact on information encoding). |
| Ashare et al., (2013)  *Effects of tolcapone on working memory and brain activity in abstinent smokers: a proof-of-concept study.* | N = 20 smokers (aged: 18-65 years), smoked at least 10 cigarettes a day for at least 6 months.  Participants completed 8 days of treatment with tolcapone and placebo | A double-blind crossover design was used to test whether tolcapone (a catechol-O-methyltransferase inhibitor) would have an effect on WM brain correlates activity and performance in abstinent smokers. (BOLD) fMRI scans were conducted whilst participants performed a WM (N-back) task after a 24 h period of abstinence | Tolcapone modestly improved accuracy of the N-back task compared to placebo. There was also lower activation in the ventromedial prefrontal cortex compared with controls. However, there were no effects in other regions of interest (such as the DLPFC, dorsal cingulate/medial prefrontal cortex, or posterior cingulate cortex). There is evidence Tolcapone led to a decrease in FMRI signalling in certain brain regions among smokers with val/val genotypes but stayed the same or increased among met allele carriers. Withdrawal, craving or mood was not affected by Tolcapone. | The study does not provide strong enough evidence to support further examination of COMT inhibitors as smoking cessation aids. |
| Ashor (2011)  *Degree of dependence influences the effects of smoking on psychomotor performance and working memory capacity.* | N = 32 adult males  N=10 Non-smokers  N=11 Light smokers  N=11 Heavy smokers | Randomised, controlled, prospective study on the effects of smoking dependence on psychomotor performance and WM capacity. The Leeds psychomotor performance test battery was used to measure choice reaction time and flicker fusion and WM capacity was measured by the N-back task. | Results revealed a significant improvement in the ascending flicker fusion test in heavy smokers compared to the other two groups. However, heavy smokers performed worse in the 3-back task compared to the other two groups. There were no significant changes in measures of descending critical flicker fusion, components of choice reaction time, and in 1-, 2- back working memory tests. | Heavy smoking increases arousal but decreases WM capacity |
| Bach et al., (2012)  *Diminished brain functional magnetic resonance imaging activation in patients on opiate maintenance despite normal spatial working memory task performance.* | N=13 Patients receiving methadone or buprenorphine (opiate maintenance)  N = 13 HC  (4 women; mean age: 37.2 years) | FMRI was used to examine visuospatial WM performance of the patients and healthy controls. | The patients demonstrated changed neuronal activation including brain regions related to WM performance and addiction. However, there was similar behavioral performance on the visuospatial WM between the two groups. | The results of the study show no impairments of visuospatial WM capacities in opiate maintenance patients. The altered neuronal activation in the areas of the brain related to WM capability may affect cognitive performance on more complex cognitive tasks. |
| Bava et al., (2010)  *Neurocognitive correlates of white matter quality in adolescent substance users.* | N=36 Alcohol and marijuana-using adolescent  N=36 HC  (aged 16-19 years) | Chronic alcohol and marijuana using participants were examined using DTI and neurocognitive tests to determine the extent of altered white matter microstructure and the consequent cognitive effects. Regions of group difference in fractional anisotropy (FA) and mean diffusivity (MD) were analysed in relation to cognitive performance | Users had lower FA (compared to controls) in brain areas related to attention, WM and processing speed (temporal areas). Users had higher FA in occipital regions, associated with WM and complex visuomotor sequencing, whereas they also had FA in anterior regions, which is negatively associated with verbal memory performance. | Results demonstrate the differing effects of white matter development on the cognition of users compared to healthy controls. Neuroadaptation may reflect additive and subtractive responses to substance use that are complicated by competing maturational processes. |
| Bell et al., (2017)  *A randomized controlled trial of cognitive remediation and work therapy in the early phase of substance use disorder recovery for older veterans: Neurocognitive and substance use outcomes* | Adult SUD participants were recovering outpatient veterans and were randomly allocated to:  N=24 Cognitive remediation therapy (CRT) + Work Therapy:  N=24 Work therapy alone | Pilot study looking into the effects of cognitive remediation therapy (CRT) as an adjunct to outpatient treatment for SUD. Neurocognitive and substance use were assessed at baseline, at the end of treatment (3 months) and after a 6-month follow up. | Baseline measures revealed high rates of cognitive impairment. The CRT and Work Therapy revealed significant changes in WM and executive function indices. Global indices of cognition showed a nonsignificant trend favoring CRT + Work Therapy. Both groups showed improvement in substance abstinence, and differences between conditions were not significant. | Results reveal that CRT improves neurocognitive and substance use disorder (SUD) outcomes. CRT was well accepted by the participants and led to significant improvements in WM and executive functions beyond that of normal cognitive recovery. The lack of a significant difference found between the conditions for SUD outcomes might be because Work Therapy obscured the benefits of CRT. |
| Bickel et al., (2011)  *Remember the future: WM training decreases delay discounting among stimulant addicts.* | N = 27 Adults in treatment for SUD  Female n =20  Male n =7  (mean age 39 years) | Randomly assigned to receive either WM training or control training according to a yoked experimental design.  Measures of delay discounting and several other cognitive behaviours were assessed pre- and post- training. WM  training was conducted as  part of the PSSCogReHab,  Psychological Software  Services Inc. package. | Rates of discounting of  delayed rewards were significantly reduced among those who received memory training but were unchanged among those who received control training. | Neurocognitive training on WM decreases delay discounting. These results offer further evidence of a functional relationship between delay discounting and WM. |
| Bogg et al., (2012)  *Cognitive control links alcohol use, trait disinhibition, and reduced cognitive capacity: Evidence for medial prefrontal cortex dysregulation during reward-seeking behavior.* | N=11 (5 male, 6 female) alcohol dependent  N=18 (10 male, 8 female) non-alcohol dependent  university students  Mean age: 20yrs | The study looked at the mPFC as a common neuro-functional marker of excessive alcohol consumption, trait disinhibition, and reduced cognitive capacity. FMRI was used to measure participants whilsts they completed the Balloon Analogue Risk Task. A weekly interview was also conducted to track alcohol consumption interview along with assessments on self-report measures of trait disinhibition and IQ, and complex span WM task. | Greater mPFC reductions during reward-seeking behaviours (successive inflation choices) were correlated with greater typical weekly alcohol consumption, greater trait disinhibition, and lower IQ. Greater increases in mPFC during reward-seeking outcomes (successive successful inflation outcomes) with greater typical weekly alcohol consumption, greater trait disinhibition, and lower IQ. No significant results were found on measure of WM. | The results reveals that mPFC activity during risk/reward appraisal and performance monitoring correlated with greater consumption of alcohol trait disinhibition, and lower IQ. |
| Brevers et al., (2014)  *Impaired decision-making under risk in individuals with alcohol dependence.* | N=30 Asymptomatic alcohol-dependent individuals  HCs: N = 30  All >18yrs | This study investigated the decision-making capabilities of individuals with alcohol dependence in terms of known probabilities (decisions under risk).This was done by measuring decision-making under ambiguity (Iowa Gambling Task) and decision-making under risk (the Cups Task and Coin Flipping Task). WM storage (digit span forward) and dual tasking (operation span task) was also tested. | Alcohol-dependent participants performed poorly on the IGT compared to controls, demonstrating poor decision-making under ambiguity. The same was seen for the Cups and Coin Flipping Tasks, demonstrating poor decision-making under risk. Alcohol-dependent participants also showed some impairments in WM (measured by the dual tasking). The degree of this impairment was related with high-risk decision-making, demonstrating a relationship between WM and risky decisions. | In times of uncertainty (both in risk and ambiguity), the study revealed that alcohol-dependent individuals are impaired in their ability to decide optimally. Furthermore, it is speculated that at least some aspects of these impairment are linked to poor WM capacities. |
| Brooks et al. (2016)  *Psychological intervention with WM training increases basal ganglia volume: A VBM study of inpatient treatment for methamphetamine use* | N=65 Meth use  N=41 TAU  N=24 CT  N=25 HC  Mean age 28yrs, all male | 4 weeks of TAU vs. CT vs.  HC baseline  CT= WM N-back task on laptop computer from 0-back to 3-back progressively difficult scaffolding for 20 daily half-hour sessions over 4 weeks. Measures: baseline & follow-up MRI scan; self- report HADS, BIS, SRQ, VAS, TMT, WM accuracy | Larger bilateral  caudate/putamen in patients who received 4 weeks of TAU  More widespread volume increases in bilateral basal ganglia (including amygdala and hippocampus) after 4 weeks of CT, which was linked to improvements in | 4 weeks of repetitive working  memory training in male patients with SUD may normalise basal ganglia circuitry structure and function. WM training may be a useful adjunct to boost standard treatment effects (e.g. increase abstinence). |
| Bustamante et al., (2011)  *Right parietal hypoactivation in a cocaine-dependent group during a verbal working memory task.* | N=15 Cocaine-dependent (mean age: 32yrs)  N=15 HC (mean age 34yrs)  All males | The study looked at the brain differences of cocaine-dependent participants and healthy controls during a verbal WM task (2-back). | The results revealed no differences in performance between groups. Nevertheless, the cocaine-dependent group demonstrated less activation in the dorsal region of the right inferior parietal cortex. No brain area was found to be overactive compared to controls. | The results of the study show reduced activation of attention-related brain areas in the cocaine-dependent men, suggesting chronic cocaine use may make such patients more susceptible to attentional deficits. |
| Campanella et al., (2013)  *Increased cortical activity in binge drinkers during working memory task: a preliminary assessment through a functional magnetic resonance imaging study.* | N=16 Binge drinkers (mean age: 21yrs) 7 females, 9 males  N=16 HC (mean age: 22yrs) 7 females, 9 males | Participants were scanned using FMRI whilst taking part in an n-back WM task, using 0-back (control) and a 2-back condition. | Even though performance was similar, higher bilateral activity was observed in the pre-supplementary motor area in binge drinkers compared with controls. Heightened activity was also seen for binge drinkers in the DLPFC, positively correlating with number of alcohol doses consumed per occasion as well as in the cerebellum, thalamus and insula while performing the WM 2-back task, also positively correlating with  the number of drinking occasions per week. | Binge drinking alcohol leads to the activation of compensatory brain regions to facilitate with normal performance. This heightened activity could indicate vulnerability towards developing substance use disorders. |
| Caspers et al., (2010)  *Effects of alcohol- and cigarette-use disorders on global and specific measures of cognition in middle-age adults.* | N = 287 (118 men and 169 women; age range: 31 - 60 years; mean age: 43.59 years) | The study looked at the effects of alcohol-and tobacco-use disorders on specific and global cognitive abilities in middle age. To do this, a neurocognitive assessment consisting of memory, executive-functioning and global cognition measures was used. The Iowa Test of Basic Skills school-achievement tests administered from third through eighth grade was used to measure baseline cognitive function. Covarying factors included: current depression symptomology, baseline cognition and medication use. | In men, lifetime alcohol- and tobacco-use disorders were not associated with cognition. In women, those with a diagnosis of tobacco dependence (according to the DSM-IV) had lower scores on measures of executive functioning and global cognition. Furthermore, women with a lifetime diagnosis of alcohol abuse (according to the DSM-IV) was associated with higher WM. | In terms of midlife cognition, the results demonstrate minor negative effects of alcohol-use disorders (especially when current consumption is small). There were more pronounced effects of cigarette-use for women who showed greater impairments in processing speed, executive function and visuospatial abilities. |
| Chanraud et al., (2010)  *Dual tasking and working memory in alcoholism: relation to frontocerebellar circuitry.* | N=17 Alcoholics: (mean age = 44 years)  N=31 Controls (mean age = 40.4 years) | Participants underwent dual WM tasks (verbal and spatial) using low (three item) or high (six item) memory loads. In addition, participants were also scanned using structural MRI to observe the amount of nodes of the frontocerebellar system. | Both groups performed equally on the verbal WM task. The alcoholic group was significantly more affected by the arithmetic distractor on the spatial WM task compared to controls. The left thalamus and left cerebellar Crus I volumes were indicators of spatial WM performance in alcoholics. On the other hand, volumes of the right middle frontal gyrus and right cerebellar Crus I were indicators of spatial WM performance with tracking interference for controls. | The brain-behaviour correlates indicate that both groups rely on the integrity of particular corticocerebellar systems to perform the verbal and spatial WM tasks, with the pattern of correlates differing between groups. The results suggest that alcoholics may make use of additional brain areas to facilitate poorer dual-task performance. |
| Chanraud et al., (2013)  *Remapping the brain to compensate for impairment in recovering alcoholics.* | N = 30 (all males)  N=15 Recovering alcoholics (mean age: 40.1 years)  N=15 HC (mean age: 47.7 years) | The study looked at whether imagining data acquired previously would meet the criteria defining “functional compensation”. Multivariate analysis was used to test how abnormal brain activation (compared to controls) predicted normal performance. | Results provide evidence for compensatory “recruitment of cerebellar-based functional networks by alcoholics”. the results indicate that “higher intrinsic cerebellar activity in alcoholics was an adequate condition for triggering task-relevant activity in the frontal cortex required for normal working memory performance.” | Alcoholics make use of brain networks to compensate for impairment in the normally recruited executive frontocerebellar loop. This abnormal brain network use meets the requirements for “compensatory”. These results can have use in therapeutic settings if compensatory responses can be brought under internal or external control in cognition. |
| Charlet et al. (2014)  *Increased neural activity during high working memory load predicts low relapse risk in alcohol dependence.* | N=40 Detoxified Alcoholic-dependent patients: (men: n = 30, women: n= 10; mean age: 18-65 years).  N=40 HC (men: n= 30, women: n = 10; age = 44.1 years) | Neural activation during a WM n-back tasks was combined with local grey matter volumes using Biological Parametric Mapping to determine whether relapse risk during a 7-month follow up period could be predicted. | Heightened activation was observed during the high WM load task (2-back) in bilateral rostral prefrontal cortex and bilateral ventrolateral prefrontal cortex in prospective abstainers compared with relapsers, and also in left/right lateral/medial premotor cortex in abstainers compared with HCs (despite equal task performance). Slight cognitive impairment was associated with increased premotor activity during performance for prospective abstainers, but not relapsers. | In terms of prospective abstainers, the results suggest that greater use of less dysfunctional executive control brain areas may suggest a resilience associated with favourable treatment outcomes. |
| Claus & Hendershot (2015)  *Moderating effect of working memory capacity on acute alcohol effects on BOLD response during inhibition and error monitoring in male heavy drinkers.* | N=17 Heavy drinking males (mean age: 26yrs) | Within-subjects design consisting of two sessions (“alcohol session” and a “control session”, whilst completing a go/no-go task in an fMRI. WM capacity was then assessed using an operation span task. | WM capacity was observed during successful response inhibition within the superior temporal gyrus and during unsuccessful inhibition in the default mode network. In the alcohol session, individuals with poorer WM capacity showed a decrease in BOLD DMN response compared to control session, whereas individuals with higher WM capacity showed an increase in BOLD response in the alcohol session compared to control session. | Decreased neural response is associated with poor WM capacity when behavioral control is required. This may lead to difficulty with response inhibition and an increased rate of unfavourable consequences from alcohol intoxication. |
| Cousjin et al., (2014a)  *Effect of baseline cannabis use and working-memory network function on changes in cannabis use in heavy cannabis users: a prospective fMRI study.* | N = 43 (age: 18-25 years)  N=34 Heavy cannabis-users  N=41 HC (non-using) | WM function networks (during an n-back task) of controls compared with heavy-cannabis users were compared using tensor independent component analysis. WM network function was also examined in relation to cannabis use and the severity of cannabis-related problems at baseline and at 6-month follow-up | There was no significant difference in performance observed between both groups. However, the utilisation of brain networks was predictive: it was found that in cannabis users, a stronger network response during the WM task was associated with an increase in weekly cannabis use. | The results suggest that heavy-cannabis users who require more effort to complete the n-back WM task, have a greater likelihood of increasing their cannabis use. Thus WM network function may be a useful way to observe the potential course and treatment outcome in cannabis users. |
| Cousjin et al., (2014b)  *Relationship between working-memory network function and substance use: a 3-year longitudinal fMRI study in heavy cannabis users and controls.* | N = 49 (age: 18-25 years at baseline)  N=22 Currently using heavy-cannabis users  N=4 Abstinent heavy-cannabis users  N=23 Controls (non-users) | 3-year longitudinal neuro-imaging (Tensor-ICA) study was conducted, looking at differences in WM network functioning over time between substance users and controls. | Cannabis-related problems remained the same for the cannabis users (compared with other substance-related problems which increased over time). At baseline measurement, groups did not differ in n-back performance and WM network functionality. Only n-back performance accuracy improved (with WM network functionality remaining the same). WM functionality was not associated with substance-use in the cannabis-user group. | Sustained moderate to heavy use of cannabis, as well as other substances (illegal psychotropic substances, nicotine and alcohol) do not alter WM network functionality. Baseline measures were also not predictors of cannabis use and related problems 3-years later. |
| Crego et al., (2010)  *Reduced anterior prefrontal cortex activation in young binge drinkers during a visual working memory task.* | N=95 Students (age: 18-20 years)  N=41 Binge-drinkers: (21 females)  N=53 Controls (26 females) | Investigation into visual WM and its neural correlates in binge drinkers. A combined event-related potential (ERP) and exact low-resolution brain electromagnetic tomography (eLORETA) study was used to assess the groups whilst they performed a pairs continuous performance task | In comparison with controls, binge drinkers showed a smaller late positive component (LPC) related to hypoactivation of the right anterior prefrontal cortex for matching stimuli (despite adequate performance). | For binge drinkers, the results appear to illustrate alternation in recognition WM functional networks as well as indicate the impairment of prefrontal cortex function at an early age. |
| Cservenka et al., (2012)  *Atypical frontal lobe activity during verbal WM in youth with a family history of alcoholism.* | N=35 Adolescents (mean age  14 yrs)  N=19 Family history of alcoholism  (Females n=6  Males n=13)  N=16 No family history of  alcoholism  (Females n=8  Males n=8) | Using functional magnetic  resonance imaging brain responses during a Verbal WM (VWM) 2-back task | Adolescents with a family  history of alcoholism had significantly slower average reaction time when making correct responses during the 2-back condition than those with no family history. In  contrast to a vigilance control condition, while covarying  for reaction time, adolescents  with a family history of  alcoholism showed less activation during VWM than  those with no family history, in multiple areas of the  prefrontal cortex (PFC), a  brain region crucial to intact  WM skills. | Even prior to heavy alcohol  use, adolescents with a family history of alcoholism show atypical executive brain functioning during VWM, and that these differences are independent of slower  WM reaction time. These abnormalities  may contribute to  vulnerability towards  developing alcohol use disorder. |
| Dean et al., (2011)  *Acute modafinil effects on attention and inhibitory control in methamphetamine-dependent humans.* | N=17 healthy subjects:(mean age: 31.1 yrs)  N=24 methamphetamine-dependent subjects: (mean age: 35.5 yrs) | Randomised, double-blind, placebo-controlled, crossover study examining the effects of modafinil (200 mg, single oral dose) on participants’ WM, inhibitory control and processing speed/attention performance. | Modafinil improved sustained attention across both groups. No other significant difference was found or other tests of cognition, However, within the methamphetamine-dependent group, modafinil had a greater effect on inhibitory control and processing speed for participants with a high baseline frequency of methamphetamine use compared with participants with a low baseline use. | The results of the study add benefit to a clinical trial that found that modafnil may be useful for methamphetamine-dependent user who use the drug frequently. |
| Duarte et al., (2012)  *Working memory deficits affect risky decision-making in methamphetamine users with attention-deficit/hyperactivity disorder.* | N=23 participants with histories of methamphetamine (MA) dependence and ADHD (mean age: n = 43 yrs)  N=25 Participants with MA dependence alone: (mean age: n = 40.2 yrs)  N=22 Controls (mean age: n = 40 yrs) | All groups completed the Iowa Gambling Task (IGT) as part of a larger neuropsychiatric research evaluation. | Participants with WM deficits in the MA + ADHD group showed the strongest inclination to choose disadvantageous vs advantageous cards in the IGT task, illustrating an interaction between ADHD, MA, and working memory. | In MA users with ADHD, WM deficits may influence risky-decision making. |
| Evans et al., (2011)  *The smoking N-back: a measure of biased cue processing at varying levels of cognitive load.* | N = 36 (aged: 18-50 yrs)  N=21 Smokers  N=15 Nonsmokers | both groups completed 1-, 2-, and 3-back versions of the Smoking N-back task. | There was no significant differences found for 1-back between groups however, smokers had less accuracy on “matched trials relative to nonmatched trials for smoking words on the 2- and 3-back tasks” compared with controls who only showed this effect in the 3-back condition. | Task complexity may influence cognitive bias. |
| Falcone et al., (2014)  *Age-related differences in working memory deficits during nicotine withdrawal.* | N=63 Smokers (aged: 18 = 65 yrs) | Participants underwent two BOLD FMRI scans while performing a visual N-back task on two separate occasions: once with smoking as usual and then after 24 hours of abstinence. | Abstinence compared with smoking resulted in less accuracy, slower correct response time and reduced brain-related BOLD signal change (in the medial frontal/cingulate gyrus and right and left dorsolateral prefrontal cortex). For smokers aged 50 years and over, abstinence effects were lessened on all measures (compared with controls). | Abstinence effects on cognitive function appear to be heightened for younger smokers, which may be valuable in looking at age differences in smoking cessation success. |
| Federico et al., (2017)  *Multifocal cognitive dysfunction in high-dose benzodiazepine users: a cross-sectional study.* | N=25 Patients admitted for high dose benzodiazepine (BZD) dependence (aged: 18–70 yrs)   - “no neurological or psychiatric comorbidity, except anxiety or depression nor concurrent alcohol or psychotropic drug dependence”.   N=26 HC (matched to patients). | Both groups underwent testing to examine verbal, visuospatial memory, working memory, attention, and executive functions. | Patients performed significantly worse on all measures compared with controls.  Some of the patients were under the influence of the BZD cumulative dose.  Anxiety and depression had a minimal influence on the cognitive tests. | The results of the study indicate that patients with high-dose BZD intake show significant changes in cognitive functioning, which should be taken into account for those who may be implicated in responsible work roles or risky situations. |
| Fernández-Serrano et al., (2010a)  *Prevalence of executive dysfunction in cocaine, heroin and alcohol users enrolled in therapeutic communities.* | N=123 Poly-substance-dependent individuals (women: n = 13; aged 18–58 years)  N=67 HC (women: n = 8; aged 18–50 years) | The prevalence of neuropsychological impairment in executive functions of polysubstance users was looked at in the study. | Results revealed a substantial prevalence of impairment in executive functioning in polysubstance users, particularly in WM, followed by fluency, shifting, planning, multi-tasking and interference. Arithmetic (Wechsler Adult Intelligence Scale, WAIS-III) showed the largest discrepancy between users and controls. | The results indicate the need for policies and treatments to direct their attention toward addressing executive deficits of the patients, which should in turn have a positive effect on treatment compliance and rehabilitation. |
| Fernández-Serrano et al., (2010b)  *Neuropsychological consequences of alcohol and drug abuse on different components of executive functions.* | N=60 Substance-dependent individuals (SDIs): (8 female;, aged 21–49 years)  N=30 HC (6 female; aged 18–49 years) | The study looked at the effects of alcohol, cocaine, heroin and cannabis effects on executive functioning in a sample of polysubstance users seeking treatment. Severity of the drug use was also considered (quantity and duration patterns). To test this, tests of WM, fluency, cognitive flexibility, decision-making, self-regulation, analogical reasoning, and inference were given to both groups | Across all tests, SDIs had significantly worse performance compared to HCs.  Results showed effects of:  - alcohol, cannabis and cocaine on verbal fluency and decision-making;  - cannabis and cocaine use on verbal WM and analogical reasoning  - duration of cocaine and heroin use on shifting  -specific effects of duration of cocaine use on inhibition measures. | The results show a negative effect of alcohol abuse on fluency and decision-making. The other drugs looked at have both specific and generalized negative effects on different executive functioning areas. |
| Fisk et al., (2011)  *Visuospatial working memory impairment in current and previous ecstasy/polydrug users.* | N=38 Ecstasy/polydrug users (men: n = 19)  N=16 previous ecstasy/polydrug users (men: n = 1)  N=52 non ecstasy users(men: n = 8) | All groups completed serial simple spatial recall and visuospatial WM tasks. | Visuospatial WM tasks impairment was observed in both current and previous ecstasy-users. However, the overall group-effect fell to just below statistical significance once confounding drug use (cannabis and cocaine) of controls was taken into account. Nevertheless, the difference between ecstasy-users and non-users demonstrated statistically significant ecstasy-related visuospatial WM deficits. | Results reveal visuospatial WM deficits for ecstasy users, particularly under conditions of high processing demand. These results are consistent with impairment arising either in posterior parietal and occipital regions or the DLPFC which come into play to assist in higher demand situations. |
| Fitzpatrick & Crowe, (2013)  *Cognitive and emotional deficits in chronic alcoholics: a role for the cerebellum?* | N=49 chronic alcoholics (males: n = 35; mean age: 53.41 yrs)  N=29 Controls (males: 16; mean age: 54.93 yrs) | Both groups underwent testing of cognitive and affective functioning, along with measurements of cerebellar ataxia (International Cooperative Ataxia Rating Scale). | Significantly worse performance was observed for the alcoholic group compared to the controls in: visuospatial and language skills, psychomotor speed, new learning and memory, executive functioning, and emotional regulation and affect processing. No differences were found between groups for immediate attention and WM capabilities. In the alcoholic group, severity of alcohol use (total number of years of heavy drinking and total amount of abstinence) were the best predictors of cognitive and emotional functioning. A relationship was found between the degree of signs of alcoholic cerebellar degeneration (ACD) and language, executive functioning, processing speed and affect processing, even after alcohol chronicity was taken into account. | Cerebellar dysfunction mediated some of the cognitive and affective deficits found in chronic alcoholics. This supports the theory of bidirectional cerebro-cerebellar circuitry underlying cognitive and affective impairments in chronic alcoholics. |
| Fridberg et al., (2013)  *Effects of working memory load, a history of conduct disorder, and sex on decision making in substance dependent individuals.* | N=158 Substance dependence (71 female),  N=72 Substance dependence and history of childhood conduct disorder (HCCD) (24 female)  N=152 Controls (84 female)  All young adults:  (age: 18-30 yrs) | Groups underwent decision making tests (the Iowa Gambling Task) with or without a WM load aimed to strain WM capacity. Outcomes were measured according to net advantageous decisions on the IGT, and preferences for infrequent- versus frequent-punishment decks. | The substance dependence and history of childhood conduct disorder men made fewer advantageous decisions on the IGT than control men without a load. For women in the substance dependence and history of childhood conduct disorder group and control men, fewer advantageous decisions was observed with the load added. | Gender differences in the effects of HCCD, substance dependence and WM load were observed on decision making on the IGT. Substance dependence and HCCD women and control men were most strained by the WM load. As the load increases, participants are inclined toward less-frequent punishments. |
| Greenstein et al., (2010)  *The separate and combined effects of nicotine and alcohol on working memory capacity in nonabstinent smokers.* | N=127 Nonabstinent daily smokers (aged: 21-52) | Study looked at the separate and combined acute effects of alcohol and nicotine on WM capacity. Participants performed the counting span task (CSPAN) after consuming either an alcohol or placebo beverage and smoking either nicotinised or denicotinised cigarettes | Smokers who smoked the nicotinised cigarettes had significantly worse results on the CSPAN task than those who smoked the denicotinized cigarettes. Women performed better than men after consuming the alcohol beverage whereas men performed better than women after the placebo beverage (even though no main effect of alcohol on WM performance was observed). The results also found no interaction between the two substances on WM performance. | Results show that nicotine causes WM impairments in nonabstinent smokers and that gender influences the role of alcohol on WM. The results do not support the idea that nicotine compensates for deterioration in WM capacity after alcohol imbibement. |
| Gunn & Finn (2013)  *Impulsivity partially mediates the association between reduced working memory capacity and alcohol problems.* | N = 474 (varied widely in severity of alcohol problems, 57% had alcohol dependence)  Young adults: | Participants underwent measures of impulsive personality, WM capacity, and alcohol problems | Results revealed that impulsivity, WM capacity and alcohol problems were all significantly related. Through structural equation models (SEMs), impulsivity was observed to partially influence the relationship between WM capacity and alcohol problems. | The results reveal correlations between impaired WM capacity and impulsivity, which may predispose individuals to alcohol problems, although the direction of this relationship is indeterminate at this point. |
| Hanson & Luciana (2010)  *Neurocognitive impairments in MDMA and other drug users: MDMA alone may not be a cognitive risk factor.* | N=52 Abstinent polydrug users  N=29 Non-user controls  (ages 18–35 years) | Neurocognitive functioning was assessed using a comprehensive neuropsychological battery and self-report measures of drug use | Polydrug users had poorer spatial span and spatial WM scores compared to controls. MDMA use amongst users was not predictive of cognitive impairment, whereas lifetime marijuana use was a significant predictor of verbal learning and memory performance. | Even though cause-effect relationships are indeterminate, the results indicate that beyond that associated with heavy drug use, moderate MDMA use does not lead to persistent dysfunction. Polydrug use may result in temporal and frontoparietal dysfunction related to dose. Marijuana use is observed to be specially responsible for certain deficits. |
| Hanson et al., (2010)  *Longitudinal study of cognition among adolescent marijuana users over three weeks of abstinence.* | N=19 Marijuana-users (with limited alcohol and other drug use)  N=21 Non-using controls  (ages 15–19 yrs). | Measures of verbal learning, verbal WM, attention and vigilance, and time estimation were assessed on 3 occasions: after 3 days, 2 weeks, and 3 weeks of substance cessation. | Compared to controls, marijuana-users were observed to have worse: verbal learning, verbal WM and attention accuracy compared to controls. After two weeks of abstinence, there were improvements in word list learning after three weeks, on verbal WM. Attention accuracy remained deficient in substance-abstinence users compared to controls throughout the three weeks, however, attention processing speed remained similar between groups over the period. | Adolescent marijuana-users were found to have worse verbal learning and verbal WM that improved during the 3 week abstinence period. Attention deficits however remained the same. Possible hippocampal, subcortical, and prefrontal cortex abnormalities may be implicated in these results. |
| Hanson et al., (2011)  *Changes in neuropsychological functioning over 10 years following adolescent substance abuse treatment.* | N=151 alcohol and other substance use disorders (A/SUD) adolescents  N=62 Controls (without A/SUD)  Adolescents (aged: 13–18 years, Mean age: 15.7; females at baseline: 46%) | A longitudinal study examined participants with and without A/SUD with neuropsychological measures across 10 years (with assessments taking place at baseline and up to 7 times after that - semi-annually). | Substance-use was related to verbal learning and memory, visuospatial memory and verbal WM, with heavier use related to poorer cognition. Heavy alcohol-use was independently related to worse measures of verbal memory over time. Substance withdrawal was related to worse verbal learning and memory scores however, substance abuse/dependence was not associated with neuropsychological performance levels. | These results indicate that substance use during adolescence may impact areas in the brain that mature later. Withdrawal symptoms from higher amounts of substance use may result in greater neuropsychological impairment, which reflect the potential neurotoxic effects of the substances. |
| Havranek et al., (2015)  *Serotonin Transporter and Tryptophan Hydroxylase Gene Variations Mediate Working Memory Deficits of Cocaine Users.* | N=126 Cocaine-users (male: 91, female: 35)  N=94 Controls (male: 68, female: 26)  Mean age: 30 yrs | Participants underwent tests measuring visuospatial, spatial, and verbal WM tasks, genotyped for the length polymorphism in the promoter region of the 5-HTT (5-HTTLPR), the variable number of tandem repeats in the second intron of the 5-HTT (VNTR In2), two single-nucleotide polymorphisms (rs4570625 and rs1386497) in the tryptophan hydroxylase-2 (TPH2) gene and quantified for peripheral 5-HTT mRNA expression in whole-blood samples. | In terms of WM, results revealed significant gene × environment relationships between 5-HT genotypes and cocaine use: the long/long (5-HTTLPR), 9+10/9+10 (VNTR In2) and C/C (TPH2 rs1386497) genotypes were risk alleles for WM dysfunction in cocaine-users. In controls, the opposite was the case in that these polymorphisms were related to improved WM performance. Furthermore, worse executive functioning in cocaine-users was related to high 5-HTT mRNA levels whereas in controls, high levels were related to increased performance. | The 5-HT system has plays a significant role in cognitive deficit progression in chronic cocaine users. Compounds which target 5-HT transmission may therefore prove effective in treating cognitive deficits resulting from chronic cocaine-use |
| Henry et al., (2012)  *Comparison of cognitive performance in methadone maintenance patients with and without current cocaine dependence.* | N=53 Methadone maintenance patients (MMP) with cocaine dependence (CD) (mean age: 42 yrs, 57% female)  N=24 MMP without CD (mean age: 47 yrs, 63% female) | Participants underwent standard battery testing on executive functioning, psychomotor performance, attention, episodic memory and WM | No significant differences were found for scores on divided attention, balance, psychomotor coordination, WM, most measures of episodic memory, or executive function. However, MMP with CD demonstrated poorer performance on psychomotor performance/attention (simple reaction time and trail-making test A) and episodic memory (higher false alarm rates on recognition memory) compared to MMP without CD. | Due to there being small between group differences between MMP with or without CD, concurrent CD (in the absence of cocaine intoxication) is unlikely to be associated with clinically significant decreases in performance in MMP. |
| Hoffmann & al’Absi (2013)  *Working memory and speed of information processing in chronic khat users: preliminary findings.* | N=32 Chronic khat-users (male: n =26; mean age: 24.3 years).  N=26 Controls (non-users) (male: n = 13; mean age: 22.9 years) | To test whether khat users have impairments in WM and speed of information processing , these two aspects were tested against controls with forward and backward digit span test (WM) and Digit Symbol Substitution Test (speed of processing). | Results suggest that long-term khat use may lead to impairments in WM (specifically on digit backwards measures of short-term/working memory) | These results support other research into the effects of SUD on users. |
| Hoffmann et al., (2015)  *The effects of acute alcohol on psychomotor, set-shifting, and working memory performance in older men and women.* | N=26 light to moderate drinkers  All adult male (55-70 yrs) | The current behavioral effects of acute low- and moderate-dose alcohol (and placebo) on participants were examined by looking at psychomotor, set-shifting, and WM performance. | Regardless of dose, women performed better on set-shifting tasks whilst men performed better on WM tasks. Furthermore, the moderate-dose group and placebo group did not differ significantly on any test. Yet the low-dose group were observed to perform better than the moderate-dose group on set shifting and WM assessments. the placebo group also performed better in the WM task, particularly for faces. There were no sex by dose interactions. | Psychomotor, set-shifting and WM performance was not significantly altered by the low and moderate alcohol conditions. Rather, the low-dose condition had a positive effect on cognitive performance. Alcohol doses did not differentially affect women and men. |
| Houben et al., (2011)  *Getting a grip on drinking behavior: training working memory to reduce alcohol abuse.* | N=48 Heavy drinkers (25 female; mean age = 44.33 years) | Participants underwent WM training or control tasks for 25 sessions over at leaat 25 days. WM and drinking behavior was assessed before and after training. | WM ability improved after the training condition and also resulted in reducing alcohol consumption for more than 1 month following the training. This was especially the case for participants with strong automatic preferences for alcohol and thus WM training may have had indirect effects on moderating impulse control. | WM may be valuable in reducing alcohol use through improving impulse control. |
| Houston et al., (2014)  *Effects of heavy drinking on executive cognitive functioning in a community sample.* | N = 560 community sample (280 men; mean age = 36.9 years, and 280 women; mean age = 35.4 years). | The participants underwent brief executive functioning testing on attentional control, cognitive flexibility, WM and response inhibition as well as quantity/frequency of alcohol and illicit drug use in the past year. | Participants with greater alcohol consumption levels showed poorer performance on executive functioning measures, including those measuring cognitive flexibility and response inhibition. This was the case even after consideration was made to other illicit drug use as well as demographic factors such as age, gender and education. | The results support previous findings on the harmful effects of alcohol consumption on executive functioning, particularly in this case on response inhibition, cognitive flexibility, and psychomotor speed. |
| Jager et al., (2010)  *Cannabis use and memory brain function in adolescent boys: a cross-sectional multicenter functional magnetic resonance imaging study.* | N-21 Abstinent but frequent cannabis–users  N=24 Non-using peers  Pooled data from the US and Netherlands (all male, age 13-19 yrs): | FMRI with a cross-sectional study was conducted looking at the effects of cannabis on adolescents’ WM and associative memory (AM) ability. WM brain activity was measured before and after rule-based learning (automatization) and AM was assessed using a pictorial hippocampal-dependent memory task. | Normal performance on both tasks was observed for cannabis users however there was heightened activity in prefrontal regions when the task was novel, with automatisation of the task reducing activity to the same level as observed in controls. There were no cannabis use related effects on AM brain regions | During novel tasks, the prefrontal cortex is overactive in cannabis-using adolescents, which may suggest a compensatory mechanism at play. Inefficient WM utilisation became apparent when continuously changing information needed to be processed and was not related to a failure in automatisation. This lends support to notions that confirm the vulnerability of the still developing frontal lobes, which is especially concerning for early substance users, such as cannabis-using adolescents. |
| Joos et al., (2013)  *Effect of modafinil on cognitive functions in alcohol dependent patients: a randomized, placebo-controlled trial.* | N=83 Alcohol dependent patients: seeking treatment: (aged: 18–60 years) | A randomised double-blind placebo-controlled trial was performed with the use of modafinil (300 mg/d) or placebo over 10 weeks. At baseline, during and after treatment, cognitive functions (digit span task, Tower of London task, Stroop task) were assessed. | Modafinil was observed to improve verbal short-term memory (number of forward digit spans) but decreases WM scores (digit span task) compared with controls. Subgroup analyses indicated that Modafinil improved WM and verbal short-term memory for patients with low WM ability at baseline There was no effect found for modafinil on the tests of planning (Tower of London task) and selective attention (Stroop task). | The results highlight the value of Modafinil for cognitive functioning enhancement in alcohol-dependent patients but also illustrate how different subgroups within the same population may be more or less benefited. There is a further need to understand the “relationship between cognitive remediation and treatment outcome in order to design targeted treatments”. |
| Kalechstein et al., (2010)  *Modafinil administration improves working memory in methamphetamine-dependent individuals who demonstrate baseline impairment.* | N=11 Methamphetamine addicted, nontreatment-seeking participants (men: n = 7; aged: 18 and 45 years) | A double-blind, placebo-controlled study measured whether a daily dose of Modafinil (400 mg) over 3 consecutive days, would better WM ability compared to baseline. | For those participants demonstrating low WM performance at baseline, significant improvement on WM measures but not on measures of episodic memory or information processing speed were observed. For those participants demonstrating high WM performance at baseline, Modafinil did not significantly affect their ability in any domain. | Results suggest that Modafinil may improve and even reverse methamphetamine-related WM impairments, especially for those with low WM performance. |
| Kalechstein et al., (2012)  *Modafinil, but not escitalopram, improves working memory and sustained attention in long-term, high-dose cocaine users.* | N=61 Cocaine-dependent individuals (aged: 18 and 55 years)  N=14 Placebo group  N=16 Modafinil 200 mg, once daily group  N=16 Escitalopram, 20 mg, once daily group  N=15 Modafinil and Escitalopram, once daily | Participants underwent baseline assessments on WM, attention/information processing and episodic memory before undergoing the trial and then being reassessed on the same measures after 5 days of inpatient care. | Modafinil was observed to significantly improve WM ability (mean n-back span, maximum n-back span) and there was also a trend toward significant improvement in visual WM (visual accuracy) and sustained attention, consistency of response time (Variability) and reduced impulsivity (Perseveration). Escitalopram was not observed to influence any measures, independently or in conjunction with Modafinil. | Modafinil improves performance on WM measures for long-term, high-dose cocaine users. This is important for the treatment of cocaine dependence as it is likely improved WM is implicated in favourable treatment outcomes. |
| Khurana et al., (2017)  *Working memory ability and early drug use progression as predictors of adolescent substance use disorders.* | N=387  community adolescents (ages 11-13) recruited in Philadelphia, USA area.  Longitudinal data from 5 waves (2005-2010) was collected with a final follow up in 2012 | Testing a neuropsychological dual circuitry model (the automatic and reflective system) of adolescent SUD  Tested was if early working memory weaknesses was associated with acting without thinking (AWT) and Delay Discounting (DD) predict SUD  Tested if early drug use progression mediates this relation  Working memory and DD and AWT were assessed at baseline  Early drug use was modelled using yearly self-report measures  SUD was established at last wave, using the DSM-5 criteria for three commonly used substances: Tabacco Alcohol, Marijuana | Weakness in WM, associated with AWT and DD which was predictive of SUD.  WM was predictive of SUD independently of early drug use and was mediated by early drug use progression. | Weak working memory in adolescents leads to reduced self-control over impulses which in turn places these individuals at risk of developing SUD. Some of this effect is mediated by early drug use progression. |
| Kopera et al., (2012)  *Cognitive functions in abstinent alcohol-dependent patients* | N=42 male abstinent Alcohol dependent (AD) patients (mean age: 44yrs)  N=32 male healthy controls (mean age: 40 yrs) | Cognitive functioning was assessed examine the effects of prolonged abstinence on performances. | Patients that were abstinent less than a year performed poorer on attentional set shifting and working memory tests compared to HC and patients with longer abstinence periods. | Multiple domains were found impaired in AD patients suggesting impairments in extensive brain networks connection brain structures. Attentional control and spatial working memory was improved in those abstinent for longer than 1 year. |
| Liang et al., (2016)  *Reduced striatal dopamine transporter density associated with working memory deficits in opioid-dependent male subjects: a SPECT study.* | N=20 opioid dependent (OD) individuals who were methamphetamine and methadone naïve (mean age: 42yrs)  N= 20 healthy controls (HC) (mean age: 41yrs) | Striatal Dopamine transporters (DATs) levels were measured using Single-photon emission computed tomography (SPECT).  Wisconsin Cars Sorting Task (WCST) was used to assess cognitive functioning. | A significant reduction of striatal DATs was found in OD participants Striatal DATs correlated with non- perseverative errors in both groups | Repeated human opioid exposure leads to a reduction in striatal DATs, which is associated with increased non- perseverative errors. non- perseverative errors on the WCST are a sensitive measure to identify working memory deficits in relation to striatal DAT reductions |
| Loughead et al., (2015)  *Working memory-related neural activity predicts future smoking relapse* | N=80 treatment seeking smokers (age: 18-65yrs) | This study examines if WM abilities predict relapse above and beyond other behavioural and cognitive measures.  2 fMRI sessions were conducted whilst completing a working memory task (N-back)  Brief counselling session and a short term quit attempt followed.  Relapse during 7 days following assessment was biochemically confirmed.  Signal from a priori established brain regions in combination with behavioural measures were used to model outcome status, which was validated by resampling techniques | Relapse was predicted by increased craving, left DLPFC and increased PCC BOLD percent change. | The combination of reduced repression of PCC and abstinence-induced decreases in left DLPFC activation may be prognostic of poor outcome of abstinence in smokers |
| Lopes et al., (2017)  *Distinct cognitive performance and patterns of drug use among early and late onset cocaine users.* | N= 103 cocaine dependent individuals (CD) inpatients divided into 2 groups  N=52 Early onset users (EOG) (mean age 29yrs, male n=45)  N=51 later onset users (LOG) (mean age 33yrs, male n=47)  N=63 healthy controls (HC) (mean age: 26yrs, males n=54) | Neuropsychological functioning was assessed using a large battery of tasks assessing for working memory, declarative memory, attention span, and general executive functioning, divided attention. | The EOG performed poorer on working memory, declarative memory, attention span, and general executive functioning compared to the HC.  The LOG had poorer divided attention and general executive functioning compared to the HC. | The deficits observed in early onset cocaine use may reflect interference of substance use with early stages of neurodevelopment. |
| Ma et al., (2014)  *Stochastic dynamic causal modeling of working memory connections in cocaine dependence.* | N=19 cocaine dependent individuals (CD)  N=14 Healthy Controls (HC) | Stochastic dynamic causal model (DCM) analysis to study effective connectivity of 7 brain regions associated to working memory pathways using fMRI | Prefrontal modality strength was largely different in CD participants compared to HC | Findings are consistent with altered cortical striatal networks related to reduced dopamine functioning |
| Mahoney et al., (2011)  *Acute, low-dose methamphetamine administration improves attention/information processing speed and working memory in methamphetamine-dependent individuals displaying poorer cognitive performance at baseline.* | N= 19 non treatment seeking methamphetamine (MA) dependent individuals (MD)  89% male | Assessing the effect of low dose MA on working memory, attention, verbal learning and memory in a MD sample  Assessments were conducted after 4 days of controlled abstinence. Assessments were conducted twice on two occasions; 1, before and after administering a dose of intravenous saline solution, followed, by 2, before and after a dose of MA (30 mg) on the following day | Overall MA exposure did not affect neurocognitive test performances  Individuals who performed relatively poor at baseline improved significantly on attention/information processing speed and working memory.  Those who performed relatively well at baseline showed decreased performances after MA administration. | MA administration may temporality improve neurocognitive functioning in poor performing MD. The use of stimulants during treatment may elevate these deficits and improve functioning. |
| Mahoney et al., (2014)  *Preliminary findings of the effects of rivastigmine, an acetylcholinesterase inhibitor, on working memory in cocaine-dependent volunteers.* | N=31 Non treatment seeking Cocaine Dependent individuals (CD)  Were assigned to three groups in a pharmacological trail  N=16 CD placebo group (mean age: 40yrs, 13% female)  N=13 CD ravastigmine 3 mg (mean age: 44yrs, 23% female)  N=12 CD rivastigmine 6 mg (mean age: 44yrs: 17% female) | Attention/information processing, was assessed at baseline (day 0) and at follow up (day 8) after participants had received rivastigmine or placebo for 7 days (day 2-8). | Rivastigmine had significant effect on working memory performance | This study shows that CD associated working memory deficits can be improved. |
| Mahoney et al., (2017)  *The relationship between premorbid IQ and neurocognitive functioning in individuals with cocaine use disorders.* | N=113 Abstinent Cocaine users (CU) divided into 3 groups based on premorbid IQ  Above average group <110  Average=90-109  Below average group>90 | Verbal episodic memory, and working memory abilities were assessed on one occasion only | Signiticant group differences were observed between the above average and average groups with the strongest magnitude between the average and below average groups | Levels of intelligence in treatment seeking CU patients should be considered at treatment admission since neurocognitive deficits are associated with treatment outcomes and below average IQ is associated with poorer cognitive performance. |
| Manning et al., (2016)  *Neurocognitive Functioning and Treatment Outcome Following Detoxification Among Asian Alcohol-Dependent Inpatients.* | N=30 Detoxified Alcohol Dependent (AD) patients  N=30 healthy controls (HC) | Cognitive functioning was assessed in recently detoxified AD in relation to treatment outcomes.    At baseline self-reported measures of every day cognitive difficulties and dys-executive symptoms were used, in combination with, working memory  Visuospatial memory, set shifting, reflection and planning impulsivity tests using CANTAB®.  At 3 months post discharge self-reported cognitive functioning and alcohol use was reassessed. | Poorer fluid intelligence working memory, set-shifting flexibility and visuospatial memory and planning/organisation was found in the AD group compared to the HC group. But not reflection impulsivity.  ¾ of the AD population was found impaired  Significant reductions in alcohol use related variables was found, however improved cognitive functioning was found only in abstainers.  No prognostic value was found on neuropsychological measures with regard to relapse at follow up | Due to the high levels of cognitive deficits found in AD inpatient populations. Cognitively demanding intervention in early abstinence may have limited impact. |
| Marvel et al., (2012)  *An fMRI investigation of cerebellar function during verbal working memory in methadone maintenance patients.* | N=5 opioid dependent (2 males, mean age: 47yrs) methadone-maintained  N= 5 matched healthy controls (2 males, mean age 45yrs) | Working memory task (item recognition task) was administered under two conditions   1. A low working memory load matching condition 2. A high working memory load ‘manipulation’ condition   Accuracy and response times were measured during fMRI | Hyperactivity was found in Substance using groups the inferior and superior cerebellum and amygdala compared to controls  Hypo activity was found in the medial and left pre frontal and pre SMA regions at a more liberal statistical threshold | Results shed light on the working memory impairments found in these populations though an association that opioid dependence is associated with disruptions in the cerebro- cerebral circuit |
| Moeller et al., (2010)  *Working memory fMRI activation in cocaine-dependent subjects: association with treatment response.* | N=19 Cocaine Dependent (CD) patients during early abstinence (treatment admission) and at discharge | Working memory task at 3 levels of difficulty were completed during fMRI assessments | Lower brain activation in putamen, cingulate gyrus, middle and superior frontal gyri, inferior frontal gyrus pars triangularis and pars opercularis, precentral gyrus, middle and superior frontal gyrus, thalamus and caudate compared to non-drug using controls  Thalamic activation was associated with treatment response | CD patients in early remission have altered frontal, striatal and thalamic activation. Specifically, thalamic activation is linked to mesocortical and mesolimbic dopamine projections, and associated with lower treatment response. |
| Moffitt et al., (2011)  *A gradient of childhood self- control predicts health, wealth, and public safety* | N=1037 Dunedin Multidisciplinary  Health and Development Study, a longitudinal study of a complete birth cohort of children born in one city in a single year, whom were followed from birth to the age of 32 y with 96%  retention | Measures of delay of  gratification, discounting, intertemporal choice tasks. Also adult health outcomes, such as substance dependence, inflammation, and metabolic abnormalities  (e.g., overweight,  hypertension, cholesterol),  because these are known  early-warning signs for costly age-related diseases and premature mortality. The study examined wealth outcomes, such as low income, single- parent child rearing, credit problems, and poor saving  habits and criminal convictions | In a cohort of 1000,  childhood self-control predicts physical health, substance dependence, personal finances, and  criminal offending outcomes, following a gradient of self- control. Effects of children's  self-control could be  disentangled from their intelligence and social class as well as from mistakes they made as adolescents. In another cohort of 500 sibling- pairs, the sibling with lower self-control had poorer outcomes, despite shared family background | Interventions addressing self- control might reduce a panoply of societal costs, save taxpayers money, and promote prosperity. |
| Morgan et al., (2010)  *Consequences of chronic ketamine self-administration upon neurocognitive function and psychological wellbeing: a 1-year longitudinal study.* | N=150 recreational users of  N-Methyl D-aspartate (NMDA or *Ketamine*)  N=30 in 5 groups  1.Frequent users  2.Infrequent users  3.Abstinent users  4.Polydrug controls  5.Non substance users | To evaluate the long term effects of Ketamine use on neuropsychological functioning  Participants were tested at baseline and at 12 month follow up | There was an 80% response rate at follow up  Neurocognitive deficits were mainly found in frequent users  Spatial working memory and pattern recognition abilities correlated negatively.  Psychological wellbeing was less in frequent users  Symptoms of depression were more severe in frequent and abstinent user groups at 12 month follow up | Frequent Ketamine use is degenerative to neurocognitive functioning and psychological wellbeing- indicating the need for more awareness amongst young people of the dangers of its use. |
| Mota et al., (2013)  *Binge drinking trajectory and neuropsychological functioning among university students: a longitudinal study.* | Cohort study, 2 year follow up examining Binge Drinking (BD)  N=89 university students at University of Santiago di Compostela  N=40 (non BD) students who do not engage in BD at all (19 male)  N= 16 initial BD, but not at follow up (EX-BD) (4 male)  N=33 BD, both at baseline and follow up (BD) (18 male)  (Mean age: 19yrs) | Executive abilities, episodic memory and working memory was assessed at two occasions (1^st^ and 3^rd^ year) | Verbal memory and monitoring difficulties were associated with BD but bot Ex-BD or healthy control students. | In line with the literature BD is associated with cognitive functions associated with the temporomesial and dorsolateral prefrontal cortex |
| [Nguyen-Louie](https://www.ncbi.nlm.nih.gov/pubmed/?term=Nguyen-Louie%20TT%5BAuthor%5D&cauthor=true&cauthor_uid=26402354) et al., (2015)  *Effects of Emerging Alcohol and Marijuana Use Behaviors on Adolescents' Neuropsychological Functioning Over Four Years.* | N=234 Longitudinal study of substance use in adolescents (12-14 yrs) | Visuospatial abilities, psychomotor speed, processing speed, verbal memory and working memory was assessed in adolescents before substance use onset and a follow up visit (years between visits M=4, SD=2)  Substances use related predictors of neuropsychological functioning was examined using regression analyses | Alcohol use days predicted poorer visual spatial ability, verbal memory outcomes  greater drug use was associated with poorer psychomotor speed.  Substance involvement did not predict processing speed and working memory performances were predicted by more alcohol use- which was an unexpected result. | Quantity and frequency of alcohol use in adolescents may have a greater association with neuropsychological deficits than previously thought. |
| Noël et al., (2011)  *Neurocognitive determinants of novelty and sensation-seeking in individuals with alcoholism.* | N= 30 alcohol dependent detoxified individuals (mean age 45.8, SD 9.5)  N=30 healthy controls (mean age 44.1, SD 8.9)  All males | The relationship between personality traits and cognitive functioning was examined comparing detoxified alcoholics (2-3 weeks detoxified) and healthy controls in a hospital setting. | Alcohol dependent participants performed poorer on gambling task and poorer on working memory abilities and response inhibition  Poor response inhibition was associated with decision making  Alcoholics showed higher levels of sensation seeking and novelty seeking compared to healthy controls | The relationship between personality traits (novelty and sensation seeking) can predict poor decision making through deficits in response inhibition. Deficits in WM are found to relate to sensation seeking. |
| Nulsen et al., (2011)  *Electrophysiological indices of altered working memory processes in long-term ecstasy users.* | N=11 Light long term Ecstasy (MDMA, *3,4-methylenedioxy-methamphetamine*) users (4 males,  Mean age 23yrs)  N=13 Polydrug users (4 males, mean age 23yrs)  N=13 Non-users  n=13 (4 males, mean age 23 yrs) | Electroencephalogram (EEG) recordings were conducted which participants completed verbal short term memory and working memory tasks (digit- forward and backward span task respectively) comparing the three groups | All groups had lower working memory capacity compared to short term memory capacity.  The biggest difference was found in the Ecstasy group who’s performance was more suppressed by processing demands compared to the other two groups | Ecstasy users show reductions in allocated cognitive resources allocated to working memory |
| Ozsoy et al., (2013)  *Hippocampal volumes and cognitive functions in adult alcoholic patients with adolescent-onset.* | N=21 Inpatients with Alcohol Use Disorder (AUD) subdivided- depending on AUD onset- in to two subgroups,  N=13 adolescent onset (mean age 43 yrs)  N=8 late onset (mean age 44yrs)  N=13 Healthy Controls (HC) (mean age 38yrs)  All male sample | Magnetic Resonance measuring Hippocampal volumes and a neuropsychological assessment battery were conducted 4 weeks of abstinence | AUD had overall smaller hippocampal volumes compared to HC  AUD performed less well on working memory, acquisition, attention and immediate memory tasks  Of the AUD individuals the adolescent onset group had smaller hippocampal volumes compared to the late onset group.  No correlations between hippocampal volumes and neuropsychological test performances were found. | Findings suggest that that hippocampal volume loss may be characteristic of adolescent onset Alcoholism rather than late onset alcoholism. |
| Park et al., (2011)  *Brain functions associated with verbal working memory tasks among young males with alcohol use disorders.* | N=11 Individuals diagnosed with Alcohol Use Disorder (AUD)  N=10 Healthy social drinkers  All males, mean age: 24yrs | Brain activity during working memory task performances (n-back task) was examined during a neuroimaging task | The social drinkers showed less activation in the right uncus compared to the AUD group  The AUD group had less activation in the precentral, left superior parietal, left superior temporal, and left cerebellar cortex during 2- back task performances compared to 0-back task performance | Individuals diagnosed with AUD show abnormal brain activity during working memory task performances.  Unfortunately, the WM accuracy was not recorded due to faulty button box in scanner. |
| Peeters et al., (2015)  *Weaknesses in executive functioning predict the initiating of adolescents' alcohol use.* | N= 534 adolescents between 12-14 years’ old  recruited from Dutch primary schools (both mainstream and special education)  69% boys | Executive functioning was examined  Alcohol use was examined  At four separate occasions over a two-year period. | WM predicted both drinking and binge drinking onset  Response inhibition only predicted onset of first drink. | The association found between executive functioning deficits in alcohol naïve individuals suggests that a weakness in executive functioning, rather than alcohol consumption, predict initiation of binge drinking.  Weakness in executive functioning precedes drinking behaviour of adolescents  Response inhibition predicts the initiation of drinking but not binge drinking |
| Pirona & Morgan (2010)  *An investigation of the subacute effects of ecstasy on neuropsychological performance, sleep and mood in regular ecstasy users.* | N=32 Recreational ecstasy users were subdivided into two groups (19 males, 13 females)  N=16 opted to use Ecstasy (experimental group: E; 10 males, mean age: 23yrs)  N=16 recreational ecstasy using group who opted not to use ecstasy (Controls, 9 males, mean age: 23yrs) | Groups were assessed on psychometric measures and neuropsychological measures on the day before and 1,2,3 days after ecstasy use. | The group that consumed ecstasy (E) did not differ from those who did not (C) on demographic variables and ecstasy use.  In the E group mood returned to baseline within 3 days and no differences were found on day 1-3 on mood measures between groups.  No effect were found on story recall, working memory, decision making and impulsivity  Measurements of the somatic marker sensitivity test at baseline and the day after ecstasy was consumed by the E group showed poorer decisions, and E group felt less sensitive to punishment | Findings suggest that previous reports of marked subacute effects of ecstasy use may have been confounded by chronic poly drug use and co-substance use |
| Potter et al., (2013)  *Cognitive function in ecstasy naive abstinent drug dependents and MDMA users.* | N=8 Clinical Group 1: Polydrug users (excl. MDMA) 6 male, 2 female: mean age 41yrs drug free for at least 6 months prior to testing  N=17 MDMA Group 2: (13 male, 4 female: mean age 34yrs)  N=10 Control Group 3: (3 male, 7 female: mean age 37yrs), no previous history of exposure to drugs | All participants were assessed using the *Cognitive Drug Research Computerised cognitive assessment system* that included a WM component and self-reported Mood and Depression assessments (Mood States, Beck Depression Inventory) | Depression, Tension/anxiety scores, and the ‘Quality of Working Memory’ factor score was higher in the in the clinical group compared to the MDMA and HC groups. The MDMA group did not differ from the HC on mood and/or cognition measures | The authors conclude that although the participants had a 6-month abstinence the cognitive deficits related to ‘heavy’ usage or the dependence on or abuse of illicit drugs are not reversed by abstinence. |
| Rapeli et al., (2012)  *Do drug treatment variables predict cognitive performance in multidrug-treated opioid-dependent patients? A regression analysis study.* | N= 104 opioid dependent patients  N=52 treated with buprenorphine (mean age= 31yrs)  N=52 treated with methadone (mean age=35yrs) | Attention, WM, visual van verbal memory tests after 6 months of treatment. | No significant difference in neuropsychological performances were found between groups, except, the simple reaction time task. Here the Buprenorphine treated patients performed better than the methadone group  10% of the variability in attention performances was associated with medication related variables  20% variance was explained in verbal memory performances by frequent substance use during the past month and treatment with more than one psychoactive drug (other than Benzodiazepine or prescription opioids) | There is an indication that multiple prescription drug use may lead to poorer cognitive outcomes in Opioid dependent inpatient populations. These results are of relevance to rehabilitation of these individuals, treatment other than the use of benzodiazepines |
| Rass et al., (2015)  *A randomized controlled trial of the effects of working memory training in methadone maintenance patients* | Methadone maintenance patients n=56  N=28 Working memory training group (WMT) (mean age: 43yrs, 16 females)  N=28 Active control group (CG) (mean age: 44yrs, 14 females) | 25 computerised working memory training sessions run in parallel with the active control group.  Drug use and cognitive outcomes were assessed before and after training. | The WMT group showed improved working memory performances on 2 of 4 tasks.  Both groups improved on 1 of 4 WM tasks.  Drug use decreased in the WMT group however no improvements on delay discounting or tasks similar to the working memory training tasks were found. | Due to the lack of working memory training effect on delayed discounting and working memory tasks similar to the training tasks this research suggests that another mechanism- such as increased distress tolerance) may be responsible for the improved drug use outcomes. |
| Rhodes & Hawk (2016)  *Smoke and mirrors: The overnight abstinence paradigm as an index of disrupted cognitive function.* | N=30 smokers attending an overnight abstinence paradigm on two separate occasions | Two assessments were conducted; a smoking visit and a non-smoking visit  Working memory, inhibition and attention were assessed each visit  Test retest of effect of smoking versus abstinence on cognitive functioning was examined | Small to medium effect sizes were found for smoking abstinence on cognitive measures  Test retest was aggregated over multiple visits (across and between pair visits), however, the test-retest effects for abstinence effects (smoking versus abstinent) on cognition were weak. | The study concludes that aggregating across multiple study visits and/or multiple measures of cognition. The patterns of poor test retest reliability for abstinence effects however suggests that the use of trait-like markers of cognitive outcomes should be applied with caution |
| [Sanvicente-Vieira](https://www.ncbi.nlm.nih.gov/pubmed/?term=Sanvicente-Vieira%20B%5BAuthor%5D&cauthor=true&cauthor_uid=27111700) et al., (2016)  *Crack-cocaine dependence and aging: effects on working memory.* | N=26 crack cocaine dependent (CRK)  N=19 healthy older adults (HO)  N=32 healthy young adults (HC)  Young adult crack cocaine dependent users all female | Working memory performances were compared between three groups whilst controlling for age effects. | Significant poorer performances were found between the CRK and HO groups compared to the HC group.  No difference was found between the HO and CRK group on WM performances. | HC and CRK participants performed similar despite controlling for age. These finding suggest that aging and crack cocaine exposure may share similar cognitive indices of cognitive decline. |
| Schweinsburg et al., (2010)  *The influence of recency of use on fMRI response during spatial working memory in adolescent marijuana users.* | N=13 Recent Marijuana users (2-7 days abstinent)  N=13 abstinent users (27-60 days abstinent)  N=18 non using controls  Ages ranged from 15-18 | Adolescents performed a n-back WM paradigm | Marijuana users showed increased activation in the bilateral insula as well as the left and medial superior prefrontal cortices, and greater activation in the right precentral gyrus was found in abstinent users whilst all participants performed similar on the task. | Recent Marijuana use disrupts results in compensatory brain activation in response to disrupted WM related neural connections, however prolonged abstinence may be associated with improvements in WM response |
| Smith et al., (2010)  *Effects of marijuana on visuospatial working memory: an fMRI study in young adults.* | N=10 Marijuana (6 males, mean age: 20yrs)  N=14 non-using youth (9 males, mean age: 20yrs)  Part of the longitudinal Ottawa Prenatal Study collecting information from individuals from birth to young adulthood | To allow for potential confounding drug exposure related variables, Prenatal drug use, current and past drug use, detailed cognitive/ behavioural performances were collected.  These variables included; parental OR infant nicotine, marijuana, caffeine and alcohol exposure.  Two groups were compared during a neuroimaging 2-back working memory task measuring blood oxygen levels in task several brain regions was examined. | In the Marijuana group the middle and frontal gyri were associated with greater activation compared to non-using controls.  The right superior temporal gyrus - normally not activated during WM tasks- was activated during task performance in the Marijuana group  After controlling for possible parental/infancy drug related confounders this study found alterations in brain activity during WM tasks in Marijuana users through recruitment of extra blood to additional brain regions. | The observed compensation for cognitive deficits through increased blood flow to additional brain regions may not be sufficient in more real life situations. This may be when deficits are observed  These neural physiological effects of Marijuana using youth is critical. |
| Sutherland et al., (2011)  *Chronic smoking, but not acute nicotine administration, modulates neural correlates of working memory.* | N=30 Nicotine dependent participants (16 female)  N=27 non- smokers (16 female)  Mean age: 33yrs | Smokers and non-smokers were scanned on two occasions whilst conducting a working memory counting paradigm.  Smokers were scanned once with a nicotine patch and once without  Non-smokers were scanned without nicotine administration on both occasions. | Across the groups the task was associated with bilateral activation in the Pre Frontal Cortex (Both lateral and medial), parietal regions, and anterior insula. Switching of attention was marked by activation in same regions but predominantly left – laterised networks.  No acute drug induced state differences were found brain activity for either task or switching effects.  Smokers did however exhibit greater tonic activation in the right anterior insula, the superior frontal cortex and the bilateral anterior pre frontal cortex though out the task. | These data suggest that nicotine dependent individuals recruit additional supervisory control operations and WM related brain activity resources during WM tasks compared to non- smokers. |
| Sweet et al., (2010)  *Effects of nicotine withdrawal on verbal working memory and associated brain response.* | N=12 Nicotine dependent participants (7 females, mean age: 39yrs) | 2- Back Working memory (WM) challenge administered during two Funtional Neuroimaging sessions focussing on the activity of the default network network (medial frontal and anterior temporal clusters).  Participants abstained from smoking prior to session and given either a nicotine patch before session or a placebo. | Amongst regions that exhibited activation during the 2-back WM task. Greater deactivation was observed in both temporal poles, and left medial gyrus.  An inverse relationship was found in the placebo condition between craving and activation in a majority of regions.  More response variability was found in activated regions in the placebo condition. | The results suggest that greater variability in neuronal responses in brain regions activated during the 2- back WM challenge are associated with craving, possibly indicating inefficient neural processing during withdrawal from nicotine in WM associated neural networks. |
| Thames et al., (2014)  *Cannabis use and neurocognitive functioning in a non-clinical sample of users.* | N=68 Recent cannabis users (mean age: 36yrs, 57% male)  N=41 past users (mean age: 51yrs, 23% male)  N=49 non-users (mean age: 35yrs, 19% male) | The effects of Cannabis use on Neurocogntive functioning was assessed  Self- reported drug use and urine drug toxicology screening, psychopathology- and neurocognitive assessments were conducted | Recent users had the poorest performances on attention/ working memory, information processing speed, and executive functioning compared to past and non- users.    No differences were found between recent and past users  Cannabis use frequency (last 4 weeks) was associated with lower global cognitive performance and all individual domains. | Even though some recovery is observed through abstinence from Cannabis use, past users still performed poorer than non-users highlighting the aversive effects of cannabis use on cognitive functioning. |
| Van Deursen et al., (2015)  *Executive Functions and Motivation as Moderators of the Relationship Between Automatic Associations and Alcohol Use in Problem Drinkers Seeking Online Help.* | N=302 problem drinkers  Mean age 52 yrs participated as of a baseline assessment before an internet intervention | Participants completed behavioural tasks assessing for valence and approach associations, working memory, response inhibition. They also completed questionnaires assessing for motivation to change and alcohol use | An interaction between working memory and valence associations was found for alcohol use, but only in those with strong motivation to change. This was not found for response inhibition or approach associations. | The results provide evidence for the intermediate role of motivation between executive and automatic processes.  More research with full range of alcohol use and motivation if recommended. |
| Verdejo-García et al., (2010)  *Impulsivity and executive functions in polysubstance-using rave attenders.* | N=25 polysubstance using Rave attenders (RvA) (60% male, mean age: 25yrs)  N= 27 drug free healthy comparisons (44% male, mean age 25yrs)  The RvA were regular users of Cannabis, Cocaine, Methamphetamine, Hallucinogens and alcohol | Drug taking interview was conducted  Impulsive behaviour Scale, delay discounting questionnaire and executive functioning (working memory, response speed, reasoning, switching, response inhibition, self-regulation decision making and emotional perception | RvA were had higher scores on impulsive measures and a greater lack of perseverance and lack of negative or positive urgency. The RvA group did not differ from the healthy control group on sensation seeking and premeditation  The RvA group had poorer inhibition/switching errors, analogical reasoning, processing speed, working memory, and decision making performances.  The RvA group performed similar to control on indices of self-regulation, reversal learning and emotion processing.  Alcohol and drug use frequency and quantity were negatively correlated with performance of executive indices and positively correlated with positive urgency. | Deficits in executive functioning and a more impulsive personality compared to non-drug taking healthy controls. These neuropsychological findings can be used to distinguish between recreational poly substance use and poly substance dependence |
| Verdejo-García et al., (2012)  *Self-regulation and treatment retention in cocaine dependent individuals: a longitudinal study.* | N=131 Spanish (Andalusia) participants with Cocaine dependence admitted to Therapeutic Communities spread over 6 districts (mean age: 34yrs, 92% male) | Working Memory, Reasoning, inhibition/switching, strategy application/multitasking and decision making was assessed within the first 30 days of treatment in (TCs)  Treatment Retention was used as the outcome measure which was calculated as period to drop out/treatment completion | Poorer executive functioning predicted treatment retention  Application and multitasking was the strongest predictor | The capacity to remain in treatment was predicted by self-regulation amongst Cocaine dependent patients. |
| Vo et al., (2014)  *Working memory impairment in cannabis- and opioid-dependent adolescents.* | N=42 youth  mean age= 18 yrs  30% female  N=19 cannabis dependent  N=23 opioid dependent | Working memory was assessed with either Wechsler Intelligence Scale for Children-IV or Adult Intelligence Scale-IV 1-2 weeks after admission to residential treatment with supervised abstinence | Significant differences in working memory performances were found between the opioid group and the cannabis group, with the opioid group performing better than the cannabis dependent patients | The finding highlight under recognized cognitive impairment in youth with Substance Use Disorders  Modification of treatment accounting for cognitive capacity are indicated to improve treatment outcomes. |
| Vollstädt-Klein et al., (2010)  *Increased activation of the ACC during a spatial working memory task in alcohol-dependence versus heavy social drinking.* | N=12 light social drinkers (5 women, 7 men, mean age: 44 yrs)  N=7 heavy social drinkers (4 women, 3 men, mean age: 52 yrs)  N=11 non abstinent alcohol dependent individuals (5 women, 6 men, mean age: 47yrs) | All participants completed a spatial working memory task and completed self-report automatic alcohol related thoughts and behaviour (obsessive compulsive drinking scale- OCDS), alcohol use in the last 90 days, and general intelligence | Behavioural performances on spatial working memory did not differ between the groups.  Alcohol dependent participants showed higher activation in the dorsal ACC (dACC) in comparison to light and heavy drinkers when controlling for general intelligence.  A positive correlation was found between the left hippocampus and the right thalamus in all participants on the OCDS. | Increased dACC activation during working memory tasks is associated with alcohol dependence. The absence of behavioural performance differences as well as the association between dACC activation and working memory indicate working memory deficits in Alcohol dependent individuals.  Low working memory capacity is linked to less self-regulated and more automatic behaviour. Additional neural activation in the left hippocampus and right thalamus in participants with higher OCDS scores may be a consequence of decreased working memory abilities due to distracting alcohol-related thoughts. |
| Vonmoos et al., (2013)  *Cognitive dysfunctions in recreational and dependent cocaine users: role of attention-deficit hyperactivity disorder, craving and early age at onset.* | N=68 Recreational Cocaine users (mean age 30yrs, 21 females)  N=30 dependent cocaine users (mean age 29yrs, 18 females)  N=68 stimulant naïve controls (mean age 33years, 8 females) | Attention, working memory, declarative memory and executive functions were examined through an extensive neuropsychological test battery | The performances of dependent cocaine users were significantly impaired on all 4 domains.  The performance of recreational users was intermediate to that of stimulant naïve participants and dependent participants, only showing significant deficits in working memory and attention  Craving, ADHD and age of onset were modulators of cognitive functioning | Cognitive deficits are found in recreational and dependent cocaine users |
| Vonmoos et al., (2014)  *Cognitive impairment in cocaine users is drug-induced but partially reversible: evidence from a longitudinal study.* | N=11 with low increased cocaine use (mean age 30yrs, 3 females)  N=8 with high increased cocaine use (mean age 34yrs, females)  N=11 with ongoing decreased cocaine use (mean age 34yrs, 3 females)  N=8 cocaine decreasers no more use (mean age 29yrs, 2 females)  N=48 cocaine naïve participants (mean age: 30yrs, 16 females)  Longitudinal Zurich Cocaine Cognition Study (ZUCO(2)St) | The study examined the changing relationship between intensity of cocaine use and cognitive functioning over a one-year period  Cognitive performance was measures at base line and 1 year follow up  Global cognitive index and the domains of attention, working memory, declarative memory and executive functioning were assessed (calculated from 13 parameters of a broad neuropsychological test battery  Cocaine use was determined from 6 month hair sample toxicology at both assessment times | Increase in cocaine use (mean +297%) was associated with deficits in working memory performances  Decreased cocaine use (mean -72%) was associated with poorer performances in attention, working memory, declarative memory and executive functioning  Individuals who ceased to use cocaine recovered completely with performances comparable to the control group  Recovery of working memory in this group was associated with age of onset- with early onset user showing less recovery | Deficits in cognitive functioning may be cocaine induced and reversible at moderate exposure  These findings indicate that cognitive changes due to cocaine use are underpinned by neuroplastic adaptations which are potentially modifiable through pharmacological or psychotherapeutical interventions |
| Wagner et al., (2013)  *Neurocognitive impairments in non-deprived smokers--results from a population-based multi-center study on smoking-related behavior.* | N=1002 smokers (mean age: 36yrs, 55% female)  N=1161 non smokers (mean age: 34yrs, 61% female) | 6 domains of cognitive functioning were compared in smokers and non-smokers.  In the smoking group the assessment battery was administered after controlled smoking of one cigarette | Significant differences were found between smokers and non-smokers on cognitive impulsivity and visual attention. This deficit was small, but significant regardless of daily cigarette consumption. Lifetime use was not correlated with cognitive functioning.  Verbal episodic memory, verbal fluency, verbal working memory, Stroop interference task did not differ between groups | The study confirms small and specific cognitive impairments in smoking individuals  The non-association between consumption of cigarettes and cognitive abilities suggests a priori deficits in cognitive abilities in smokers |
| Wardell et al., (2016)  *Impulsivity, working memory, and impaired control over alcohol: A latent variable analysis.* | N=300 young heavy drinkers (n=159 women, mean age 20yrs) | Self-report measures of alcohol use, impairments of its use and alcohol related problems  Digit Span Test to assess working memory | Response Impulsivity predicted impaired control over alcohol use and problems related to alcohol use  Reflection impulsivity predicted heavy drinking frequency  Response impulsivity was indirectly associated to alcohol problems by mediating heavy drinking frequency  Reflection Impulsivity was indirectly associated to alcohol problems through impaired control  Sensation seeking and working memory were not associated to these alcohol use variables, and no moderating role was found. | Impaired control plays a specific role in alcohol related problems through response Impulsivity but not reflection impulsivity and the role of WM is questioned. |
| Wesnes et al., (2013)  *Effects of nicotine withdrawal on cognition in a clinical trial setting.* | N=30 healthy smoking male volunteers (aged 18-56yrs)  N=10 to placebo on days 1–7 (period 1) and placebo on days 8–14 (period 2)  N=20 to placebo on days 1–7 and experimental drug (*n* = 20) on days 8–14 | CDR system tests on a PC of cognitive function including simple reaction time, digit vigilance, choice reaction time, spatial working memory, numeric working memory, immediate word recall, delayed word recall, word recognition and picture recognition. | Smoking withdrawal disrupted aspects of recognition, attention and episodic verbal recall | Study shows that withdrawal from smoking is associated with deficits in cognitive functioning |
| Winward et al., (2014)  *Heavy alcohol use, marijuana use, and concomitant use by adolescents are associated with unique and shared cognitive decrements.* | Adolescents (age 16-18)  N= 24 adolescents with episodes of heavy drinking (HED)  N=20 who use Marijuana (MJ), both MJ and HED (MJ/HED) and N=55 healthy controls (HC) | A cognitive test battery was administered following a 4-week period of monitored abstinence | The HED group showed poorer cognitive flexibility, verbal recall, semantic clustering and reading skills compared to the HC  The MJ group psychomotor speed, cued verbal memory and inhibition task accuracy  Comparable to the HED group, the MJ/HED group showed deficits on verbal recall and cognitive flexibility, relative to the HC.  Comparable to the  MJ group the MJ/HED group showed poorer inhibition task accuracy relative to the HC.  Unique to the MJ/HED group was poorer Working Memory abilities relative to HC  Lifetime use and onset of Marijuana use and lifetime and withdrawal symptoms of alcohol, correlated with poorer test performances | Concomitant use of both alcohol and marijuana use and the heavy use of these substances are associated with poorer cognitive functioning outcomes.  These separate substances appear to be linked to specific performances deficits in separate domains. |
| Yan et al., (2014)  *Working memory and affective decision-making in addiction: a neurocognitive comparison between heroin addicts, pathological gamblers and healthy controls.* | N=58 heroin dependent (HD) but abstinent (mean age 36yrs)  N=58 pathological gamblers (mean age 36yrs)  N=60 Healthy Controls (mean age 34yrs)  All male | Working memory was assessed with the Self ordered pointing test (SOPT), decision making was assessed with the Iowa Gambling Task (IGT) | HD individuals performed worse on the IGT and SOPT compared to healthy controls.  Pathological gamblers performed worse on the IGT but not the SOPT compared to the healthy control group.  A negative correlation was found between Heroin use and working memory and decision making.  Pathological gambling was not correlated to decision making and working memory | Working memory deficits in heroin dependence were not found in pathological gambler, potentially indicating substance related harmful effect. Decision making was found in both heroin and pathological gamblers potentially representing an underlying vulnerability to addiction. |
| Zeng et al., (2013)  *Impulsivity, cognitive function, and their relationship in heroin-dependent individuals.* | N= 86 abstinent heroin dependent participants (n=45 male, mean age 28yrs)  N=88 age and gender non drug using, matched controls (n=43 male, mean age 2yrs) | Self-report Impulsivity and computerised cognitive functioning assessments | Greater deficits in self-reported motor impulsivity and experience seeking in the patient group. Self-reported impulsivity was not correlated with cognitive functioning. Greater functional deficits were found in impulsivity and distractibility but not working memory or decision making | The results suggest underlying brain related factors in heroin dependence which have implications for its treatment |
| Zhong et al., (2016)  *The cognitive impairments and psychological wellbeing of methamphetamine dependent patients compared with health controls.* | N= 54 abstinent (6 months) methamphetamine dependent individuals (mean age: 34yrs, 40 male)  N=58 Healthy Controls (mean age 31yrs, 37 male) | Participants completed psychological wellbeing scales and the *CogState* Battery 6 cognitive domains twice during 6 months abstinence (at 3 month intervals) | Impaired verbal memory, problem solving and social emotional cognition improved after 6 month abstinence  Cognitive impairments related to first drug use, social cognition and quality of life  MA dependency is related to overall cognitive impairments and poor psychological wellbeing | MA dependence is related to cognitive impairment, of which impaired verbal memory, problem solving and social emotional cognition recover at 6 months abstinence, concluding that improved cognitive functioning in MA dependence should be considered an important component of treatment. |

SA=South Africa; UK=United Kingdom; US= United States; SWE=Sweden; NE=The Netherlands; HC=Healthy Controls; MA=Methamphetamine; TAU=Treatment as Usual; CT=Cognitive Training; WM=WM; MRI=Magnetic Resonance Imaging; HADS=Hospital Anxiety and Depression Scale; BIS=Barratt Impulsivity Scale; SRQ=Self Regulation Questionnaire; VAS=Visual Analogue Scale; TMT=Trail Making Task; IAPS=International Affective Picture System; RAN=Restricting Anorexia Nervosa; ADHD=Attention Deficit Hyperactivity Disorder; EDNOS=eating disorder not otherwise specified; BDNF=Brain Derived Neurotropic Factor; SNP=Single Nucleotide Polymorphism; SMA=Supplementary Motor Area; DLPFC=dorsolateral prefrontal cortex; m=male; f=female; EDI=Eating Disorder Inventory; ACC=Anterior Cingulate Cortex
