# Supplementary material for: The Role of Working Memory for Cognitive Control in Anorexia Nervosa versus Substance Use Disorder
Source: Front Psychol. 2017 Sep 22;8:1651. doi: 10.3389/fpsyg.2017.01651 (PMC5615794; doi:10.3389/fpsyg.2017.01651)
Supplement: Supplementary file 3 [file Table_3.PDF]

PRISMA 2009 Flow Diagram

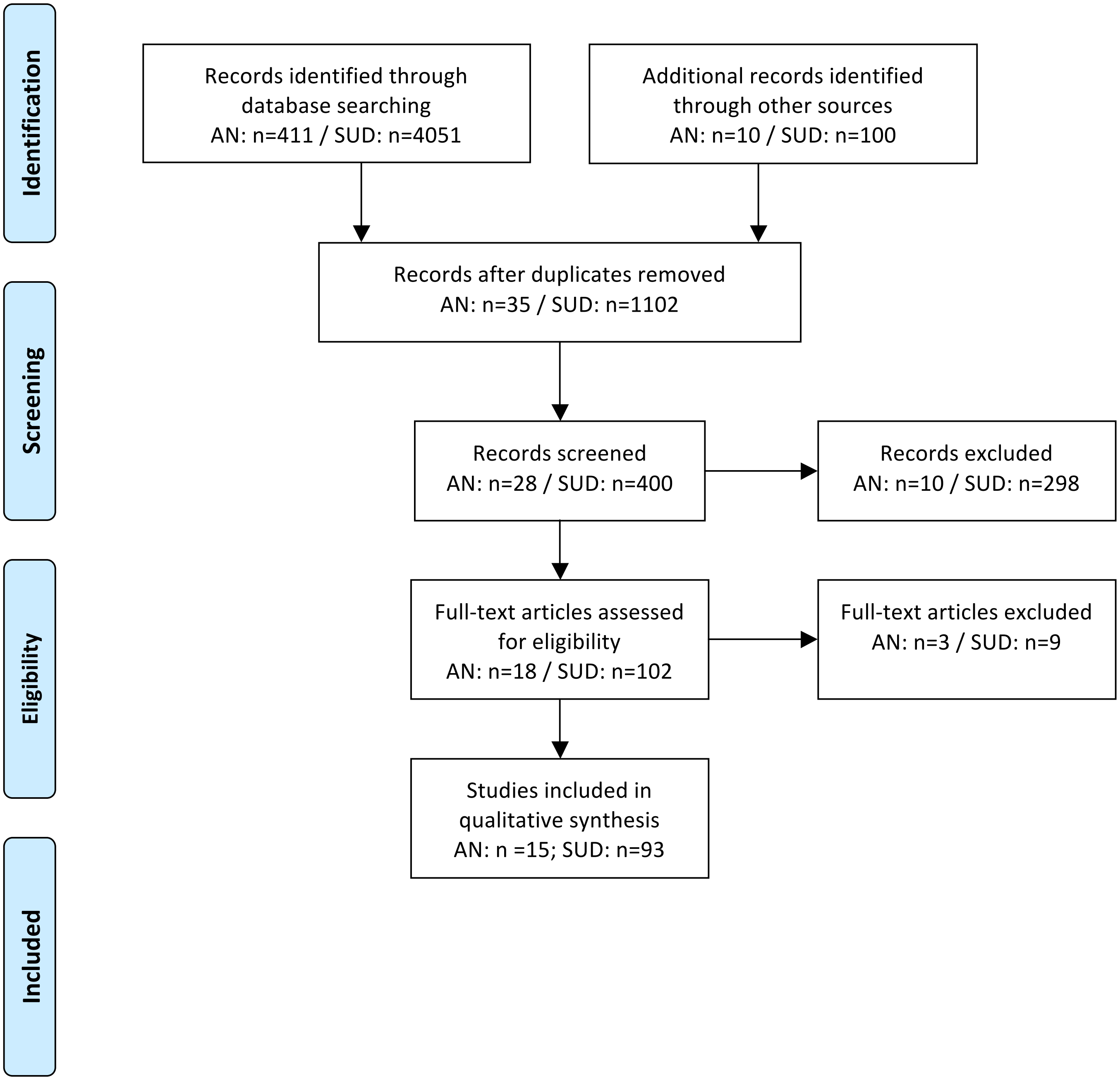

From: Moher D, Liberati A, Tetzlaff J, Altman DG, The PRISMA Group (2009). *P*referred *R*eporting *I*tems for *S*ystematic Reviews and *M*eta-*A*nalyses: The PRISMA Statement. PLoS Med 6(7): e1000097. doi:10.1371/journal.pmed1000097

For more information, visit www.prisma-statement.org.
